# Soil bacterial responses to experimental warming and drought across winter wheat growth stages in the North China Plain

**DOI:** 10.3389/fpls.2025.1637476

**Published:** 2025-12-16

**Authors:** Djifa Fidele Kpalari, Yuanyuan Fu, Sen Li, Hui Cao, Rakhwe Kama, Abdoul Kader Mounkaila Hamani, Junming Liu, Shoutian Ma, Dongxue Lv, Yang Gao

**Affiliations:** 1 Institute of Farmland Irrigation, Chinese Academy of Agricultural Sciences/Key Laboratory of Crop Water Use and Regulation, Ministry of Agriculture and Rural Affairs, Xinxiang, China; 2 College of Natural Resources and Environment, South China Agricultural University, Guangzhou, China; 3 College of Tropical Crops, Hainan University, Haikou, China; 4 School of Hydraulic and Civil Engineering, Ludong University, Yantai, Shandong, China; 5 College of Water Hydraulic and Architectural Engineering, Tarim University, Alar, China

**Keywords:** warming, drought, rhizosphere bacterial community, root exudates, wheat growth stage

## Abstract

**Introduction:**

While climate change alters the balance of the terrestrial ecosystems, the impact on the soil bacterial community remains poorly understood. A field experiment was conducted to assess the effects of warming, drought, and their combination on the soil bacterial community at different growth stages of winter wheat.

**Methods:**

Four treatments were defined for this study: warming at 1.5°C combined with full irrigation (TWS) and deficit irrigation (TWD), then ambient temperature combined with full irrigation (TNS) and deficit irrigation (TND).

**Results:**

TWS, unlike TND, promoted nitrogen availability for plants and root exudation. The abundance and diversity of the bacterial community were more responsive to different climatic stresses at the jointing stage than at other growth stages. *Chloroflexi*, *Firmicutes*, and *Bacteroidota* were positively correlated with soil inorganic nitrogen, the root total organic carbon (TOC), and negatively correlated with available phosphorus (AP), available potassium (AK), soil organic carbon (SOC) under TND, while an opposite trend was observed with *Actinobacteria* and *Proteobacteria*. Furthermore, under TWS, *Bacteroidota*, unlike *Actinobacteria*, was positively correlated with NH_4_^+^, NO_3_^-^, TOC, and negatively correlated with AP, and SOC. The bacterial community network feature values were higher under TWD and lower under TNS.

**Conclusion:**

These results indicate that the sensitivity of the rhizosphere bacterial community to the different climatic stresses varies according to the growth stage, and that the community is particularly more responsive at the jointing stage than at the later stages.

## 1 Introduction

Soil microbes are living organisms that perform multiple complex functions within terrestrial ecosystems. Although some of these organisms can harm plants, they play a crucial role in plant protection and growth. Microbes such as plant growth-promoting rhizobacteria (PGPR) accelerate plant growth by improving nutritional capacity and resistance to environmental stresses and by producing growth-stimulating hormones (Lyu et al., 2019; Verma et al., 2019; Vocciante et al., 2022), which positively affect crop productivity (Kumar and Verma, 2019). The soil microbial community maintains soil quality and facilitates nutrient availability to crops by participating in nutrient mineralization processes (Rashid et al., 2019; Schloter et al., 2018). Indeed, several studies have shown the fundamental function of microorganisms in transforming organic matter, nitrogen, and phosphorus in soil (Duan et al., 2022; Yang et al., 2022; Li et al., 2021). Other studies have highlighted the importance of soil microorganisms in regulating greenhouse gas emissions such as carbon dioxide, methane, and nitrous oxide (Arunrat et al., 2025a; You et al., 2022; Kpalari et al., 2023). However, various environmental stresses, such as extreme climatic events, compromise the efficient functioning of the community (De Vries et al., 2018; Mekala and Polepongu, 2019).

The impact of climate change on living organisms has been a major concern for researchers in recent decades. This change manifests in various anomalies, including drought and rising atmospheric temperatures (Fawzy et al., 2020; Marengo et al., 2017). Efforts have been made in several areas to stabilize the increase in atmospheric temperature at 1.5 °C compared with pre-industrialization times (Hoegh-Guldberg et al., 2019; Masson-Delmotte et al., 2018). However, research continues to report a steady rise in temperature in different regions across the globe (Arnell et al., 2019; Hashimoto, 2019; Lindsey and Dahlman, 2020), and this rise could reach 4 °C by the end of this century if no action is taken to limit excessive greenhouse gas emissions (Wang et al., 2018). The increase in atmospheric temperature intensifies the risk of drought or exacerbates it in regions where both cooccur (Diffenbaugh et al., 2015; Vicente-Serrano et al., 2020). Xu et al. (2019) reported that, globally, a 1.5 °C increase in atmospheric temperature is likely to increase drought frequency by 36% and its duration by 15%.

Several studies have attempted to document the influence of warming on soil microorganisms. Previous research has suggested that an increase in atmospheric temperature has beneficial impacts on the diversity of soil microbial community (Fang et al., 2021; Wang et al., 2020; Yu et al., 2021a). However, this impact depends on several parameters, including the duration of the stress (Wu et al., 2022; Zheng et al., 2020) and the soil layer depth considered (Fu et al., 2023; Zhang et al., 2015). Increased temperature modifies the abundance and composition of the soil microbial community by increasing the proportion of specific bacterial phyla, such as *Proteobacteria* and *Actinobacteria*, to the detriment of others (Che et al., 2022; Li et al., 2023b). Previous studies have reported positive impacts of this climatic phenomenon on soil microbial biomass (Chen et al., 2021; Ma et al., 2019) and according to Chen et al. (2021), the response of soil microbial carbon and nitrogen biomass to this thermal stress is not uniform. However, this biomass undergoes significant long-term decreases as the duration of warming increases (Xu and Yuan, 2017).

Drought is becoming an increasingly common phenomenon in different regions of the world (Dai et al., 2018), and its influence does not spare living soil organisms. Studies have shown that the unavailability or decrease in the quantity of water in the soil is likely to induce various stresses in the soil microbial community, which may result in the either death or biological adaptation (Bogati and Walczak, 2022; Schimel, 2018). Unlike global warming, drought alters the diversity (Preece et al., 2019; Siebielec et al., 2020) and biomass of the soil microbial community (Xu et al., 2020; Sun et al., 2020c). This stress disrupts the abundance and composition of the bacterial community by modifying the relative proportion of the different bacterial phyla composing it (Preece et al., 2019; Veach and Zeglin, 2020; Ochoa-Hueso et al., 2018; Kpalari et al., 2023). Bu et al. (2018) reported an increase in the relative proportions of *Acidobacteria* and a decrease in that of *Proteobacteria* under drought conditions. Other studies have also shown that water deficit reduces soil microbial diversity (Canarini et al., 2021) while destabilizing the community complexity (De Vries et al., 2018).

Temperature and precipitation are among the most influential climatic factors for the soil microbial community (Li et al., 2018). The combined impact of extreme climatic events such as drought and warming reduces the community’s abundance, composition, and functional properties (Von Rein et al., 2016; Yang et al., 2021a; Li et al., 2018). According to Sheik et al. (2011), the combination of these two climatic anomalies is capable of inducing a 50-80% reduction in the size of the soil microbial population. However, most previous studies considered only the crop maturity stage, and the impacts of climate change on the community at the different stages of crop growth remain poorly documented.

Winter wheat is one of the most widely cultivated crops in China, and its productivity depends on the various stresses it is subjected to during the different stages of its growth (Feng et al., 2020; Monteleone et al., 2023). However, this crop is highly sensitive to extreme weather events (Chandio et al., 2023; Sun et al., 2024; Shoukat et al., 2024). The main objective of this study was to simulate the impacts of different climate scenarios on the microbial community in the rhizosphere of winter wheat at different growth stages. The present study aims to (i) investigate the individual and combined effects of different climatic phenomena on the soil bacterial community and (ii) explain the community’s response to climatic stresses at different growth stages of winter wheat. We hypothesize that the response of the bacterial community to climatic stress depends on the plant growth stage.

## 2 Materials and methods

### 2.1 Site description

The experiment was conducted from October 2022 to June 2023 at the Qiliying Experimental Station, Institute of Farmland Irrigation of Chinese Academy of Agricultural Science (35.09^°^N, 113.48^°^E, and altitude 81 m). The trial was installed in lysimeters under the rain shelter. There were 24 lysimeters, and each measured 3.33 m×2.0 m in size, with a 1.8 m soil depth. The soil was sandy loam, with a field capacity (FC) of 29% (mass basis), bulk density of 1.45 g cm^3^, total nitrogen of 72.57 mg kg^-1^, available phosphorous of 45.25 mg kg^-1^, soil organic matter of 8.98 mg kg^-1^, soil organic carbon of 5.21 mg kg^-1^ and pH (H_2_O) of 8.54. The trial area has a continental monsoon climate with an average annual rainfall of 548 mm, an average yearly temperature of 14.5 °C, and a sunlight duration of 2398.8 hours.

### 2.2 Experimental design

**Figure 1:**
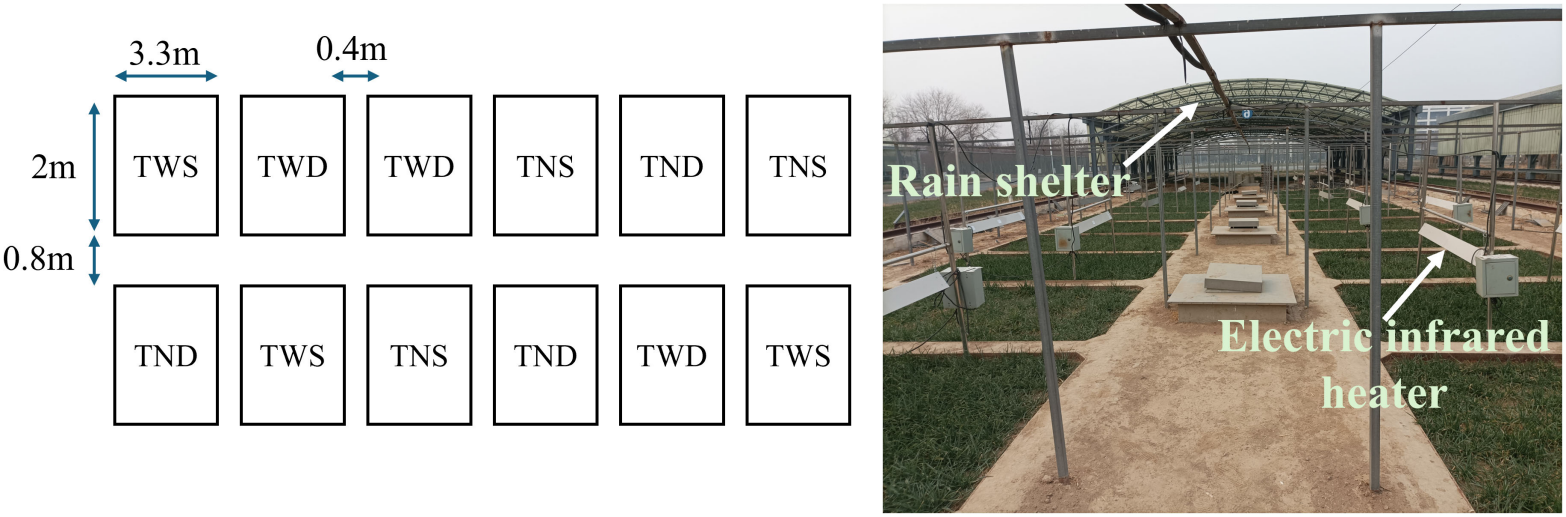
Experimental design.

The experimental design was identical to that used by Li et al. (2023a) (**Figure 1**). A randomized complete block was designed with two temperature levels and two irrigation regimes. Four treatments were defined for this study: warming combined with full irrigation (TWS) and deficit irrigation (TWD), then ambient temperature combined with full irrigation (TNS) and deficit irrigation (TND). The control treatment was TNS, and all the treatments were replicated three times. The two temperature levels were set as warming at 1.5˚C and non-warming, and the two soil moisture levels as full water supply (irrigation rate of 45 mm) and deficit water supply (irrigation rate of 33 mm). The electric infrared heater (model MRM2420, Kalglo Electronics Co., Inc., PA, USA) was used to warm the air. An infrared heater is made of an iron bracket, a far-infrared heating black-body tube (length of 1.8 m and diameter of 1.8 cm) with a power of 2,000 W, and a white stainless steel reflecting cover with dimensions of 2 m×0.2 m. Before sowing, the bracket was fixed in the soil, and an iron support held the far-infrared heating tube in place. To increase the temperature to approximately 1.5 °C, the height of the heating tube was adjusted regularly, and a manual thermometer was used to check the temperature above the canopy every morning at 8 a.m. During the winter wheat growing season, a 24-hour continuous warming mode was employed, and the effective warming area was 4 m^2^. A lampshade was also provided for the non-warming treatments to control and lower the error of test factors.

The winter wheat (*Triticum aestivum* L.) variety Zhoumai-22 was used in this study. Water is applied by drip irrigation when the average soil moisture between 0–80 cm depth of soil falls to 60%-65% of the field capacity. As regards fertilization, urea (46% N) calcium superphosphate (16% P_2_O_5_), and potassium sulfate (52% K_2_O) were applied to the crops at doses of 240 kg/ha N, 120 kg/ha P, and 120 kg/ha K based on the fertilization formulas recommended in our study area (Liu et al., 2024b).

### 2.3 Soil sample collection

Three soil samples were collected per treatment for the soil chemical properties analysis, and three other soil samples from the rhizosphere were collected for microbial analysis. The samples were taken at three growth stages of winter wheat: jointing, flowering, and grain-filling. For each plot, soil samples were taken between 0–10 cm soil depth and used to measure the soil acidity (pH), soil organic matter (SOM), soil organic carbon (SOC), available phosphorus (AP), available potassium (AK), total nitrogen (TN) and soil inorganic nitrogen (NO_3_^-^ and NH_4_^+^). Soil within 2 mm of the root surface was considered rhizosphere soil in this study (Chen et al., 2019). After gently shaking the roots to remove loosely adhering soil clumps, rhizosphere soil samples were carefully collected by brushing the roots to remove any remaining soil and stored at -80 °C before being sent to the Shanghai Majorbio Laboratory for soil microbial community structure analysis.

### 2.4 Soil sample analysis

AP concentrations were determined calorimetrically (ammonium vanadate/molybdate) using spectrophotometry (540 nm, Hitachi U-2900 Double-Beam UV-Visible Spectrophotometer), while AK concentrations were measured directly in the CAL extract via flame spectrometry (Eppendorfer ELEX 6361) (Reimer et al., 2020). TN was determined by extracting soil samples for an hour using 2 M KCl (1:10) and filtering the resulting extracts using a 0.45 mm membrane. The soil pH was determined in distilled deionized water following standard procedures for soil pH measurement as described by Kome et al. (2018), SOM, and SOC by wet oxidation method (Walkley and Black, 1934). Soil NO_3_^-^ and NH_4_^+^ content were determined with a continuous flow auto-analyzer as described by Ning et al. (2019).

### 2.5 DNA extraction and sequencing

DNA extraction was performed in 0.5 g of soil using E.Z.N.A Soil DNA Kit (Omega Bio-tek, Norcross, GA, USA), and purity was assessed using ultraviolet–visible spectrophotometer (Thermo Scientific, Wilmington, NC, United States). DNA quality was checked by 1% agarose gel electrophoresis. The hypervariable region V3-V4 of the bacterial 16S rRNA was amplified using specific primers (338F: 5’-ACTCCTACGGGAGGCAGCAG-3’; 806R: 5’-GGACTACHVGGGTWTCTAAT-3’) (GeneAmp 9700, ABI, USA). The PCR amplification was performed using the following process: 27 cycles of denaturation at 95 °C for 30 s, annealing at 55 °C for 30 s, extension at 72 °C for 30 s, and final extension at 72 °C for 10 min. The PCR mixtures contain 4 µl of 5 × TransStart FastPfu buffer, 2 µl of 2.5-mM deoxynucleoside triphosphates (dNTP), 0.8 µl of forward primer (5 µM), reverse primer (5 µM) 0.8 µl, 0.4 µl of TransStart FastPfu DNA Polymerase, and 10 ng of template DNA. The PCR products were extracted from a 2% agarose gel and quantified using a Quantus Fluorometer system (Promega, USA). Purified amplicons were pooled in equimolar and paired-end sequenced on a MiSeq platform (Illumina, San Diego, CA, USA) by Majorbio Bio-Pharm Technology Co. Ltd. (Shanghai, China).

### 2.6 Root exudation collection and analysis

Root exudates were collected at the jointing, flowering, and grain-filling stages using a modified culture-based cupping system developed specifically for collecting root exudates in the field (Phillips et al., 2008). Plants were selected randomly from each plot, and a hole was dug under each plant to isolate a few fine-branched roots of similar length and branching (2 mm average diameter with laterals) between 0–10 cm soil depth. The isolated roots were then carefully cleaned of sand and rinsed with deionized water (Ulrich et al., 2022). The roots were then placed in tubes containing sterile 2 mm diameter glass beads to simulate soil porosity and mechanical impedance in a carbon-free matrix. The beads covering the roots were moistened with a dilute sterile carbon-free nutrient mixture (0.5 mM NH_4_NO_3_, 0.1 mM KH_2_PO_4_, 0.2 mM K_2_SO_4_, 0.2 mM MgSO_4_, 0.3 mM CaCl_2_) used as a culture medium. The assembly was covered with aluminum foil and re-entered into the soil to minimize photolytic degradation of the root acids (Liu et al., 2022a; Phillips et al., 2008). 48 hours after the exudate collection device was installed, the roots were rinsed three times, and the nutrient medium was renewed. The nutrient medium containing root exudates was collected 24 hours after the previous operation and filtered to remove cellular debris (Nakayama and Tateno, 2018; Leuschner et al., 2022).

The total organic carbon (TOC) contained in the root exudates was measured using a model TOC-V CHS/CSN total organic carbon analyzer (Shimadzu Corporation, Kyoto, Japan) (Karst et al., 2017; Staszel et al., 2022). The roots were then scanned and weighed to adjust the TOC analysis results.

### 2.7 Data analysis

The Majorbio laboratory’s online platform (www.majorbio.com, accessed on 10 August 2023), R software (version 4.3.1), and Gephi software (version 0.10) were used to analyze the data and draw the graphs. A one-way analysis of variance (ANOVA), followed by Tukey’s honest *post-hoc* test, was used to study the effect of different climatic stresses on the chemical parameters and alpha-diversity indices of the soil bacterial community. Principal coordinate analysis (PCoA), followed by the similarity test (ANOSIM) and permutational multivariate analysis of variance (ADONIS), was used to analyze the difference in bacterial community composition under the different treatments. This method is faster for large datasets and allows for a direct interpretation of distances and explained variance compared to other methods, such as NMDS and DCA (Ramette, 2007). All tests were performed at the 5% significance level.

The data on the relative abundance of community ASVs were used to construct co-occurrence networks (relative abundance > 40%, defined after a sensitivity analysis). The data was prepared with Spearman correlation between ASVs and random matrix theory using the igraph package to maintain comparability with previous studies (Khan et al., 2025; Shen et al., 2023). The P-values were adjusted using the Benjamini and Hochberg false discovery rates (P < 0.01), and the similarity threshold for the networks was set to 0.8. The visualization of the networks was realized using Gephi software (version 0.10) with the Fruchterman-Reingold algorithm.

Partial least squares (PLS) was conducted using the plspm package to evaluate the effect of climate, soil, and different growth stages of winter wheat on the microbial community diversity. The Pearson correlation test assessed the relationships between the abundance of bacterial phyla and environmental parameters. Redundancy analysis (RDA) with the vegan package and heatmap with the ComplexHeatmap package were used to visualize this relationship.

## 3 Results

### 3.1 Soil chemical properties and total organic carbon of root exudats under different climatic conditions

The quantities of soil nutrients and organic carbon of root exudates under the different treatments are presented in **Table 1**. The test of one-way ANOVA showed a significant influence of the different climatic conditions on soil chemical properties (p<0.05). Compared to TNS, TWS significantly increased the available nitrogen content of soil at the jointing and flowering stages but not at the grain-filling stage. In contrast, TND decreased the quantity of available nitrogen at the jointing stage and an increase at the flowering and grain-filling stages. The quantities of the major nutrients under TWD were relatively similar to those under control treatment (TNS) at all winter wheat developmental stages.

**Table 1:** Soil chemical properties and total organic carbon of root exudation under different climatic conditions.

Treatments		NH_4_^+^	NO_3_^-^	AP	AK	pH	TN	SOC	SOM	TOC
J	TWS	15.99 a	125.78 a	26.53 a	107.08 b	8.44 a	0.51 b	5.58 a	9.56 a	11.74 a
	TWD	9.31 b	62.82 c	15.67 b	101.77 b	8.42 a	0.47 b	4.75 b	8.22 b	8.9 b
	TNS	9.54 b	76.58 c	20.99 ab	148.25 a	8.29 a	0.58 a	4.97 b	8.4 b	9.01 b
	TND	12.07 b	102.08 b	18.89 b	103.1 b	8.25 a	0.51 b	5.4 a	9.3 a	9.85 b
	*P-value*	*0.001*	*0.001*	*0.005*	*0.001*	*0.058*	*0.004*	*0.001*	*0.003*	*0.01*
F	TWS	5.14 ab	82.32 a	33.51 a	117.58 b	8.28 a	0.64 b	6.32 a	10.9 a	7.81 a
	TWD	3.87 b	83.56 a	30.22 a	154.24 a	8.25 a	0.57 c	6.16 a	10.61 a	3.39 b
	TNS	4.27 b	63.36 b	28.89 a	118.9 b	8.34 a	0.7 a	5.51 b	9.46 b	3.13 b
	TND	5.66 a	86.93 a	29.91 a	116.37 b	8.27 a	0.58 bc	5.91 ab	10.19 a	4.52 b
	*P-value*	*0.013*	*0.001*	*0.14*	*0.004*	*0.394*	*0.001*	*0.009*	*0.002*	*0.015*
G	TWS	4.74 ab	82.5 b	29.35 a	109.35 b	8.41 a	0.61 a	6.02 a	10.49 a	2.21 a
	TWD	4.95 a	71.41 b	26.08 b	139.98 a	8.32 ab	0.53 a	5.84 ab	10.33 a	2.22 a
	TNS	3.36 b	61.36 b	25.67 b	111.79 b	8.32 ab	0.65 a	5.58 ab	9.96 a	2.1 a
	TND	5.52 a	111.18 a	24.77 b	102.79 b	8.17 b	0.53 a	5.14 b	9.21 b	1.68 b
	*P-value*	*0.015*	*0.001*	*0.001*	*0.001*	*0.054*	*0.067*	*0.056*	*0.005*	*0.11*

Means followed by the same letter within the same column for each growth stage are not significantly different at α = 0.05, according to Tukey’s test. TWS warming + full irrigation; TWD warming + deficit irrigation; TNS ambient temperature + full irrigation; TND ambient temperature and deficit irrigation; J jointing stage; F flowering stage; G grain filling stage. NH_4_^+^ ammonium; NO_3_^−^ nitrate; AP available phosphorous (mg kg^-1^); AK available potassium (mg kg^-1^); pH soil acidity; TN total nitrogen (mg kg^-1^); SOC soil organic carbon (g Kg^-1^); SOM soil organic matter (g Kg^-1^); TOC total organic carbon of root exudation (mg C g^-1^ root dry weight).

The highest quantities of SOC and TOC were obtained under TWS at all stages. Under TWD, the quantities of these three soil parameters were identical to those under the control treatment at all stages except at the flowering stage, where an increase in SOC was observed (**Table 1**). The quantity of TOC under TND was also identical to that under the control treatment at all stages except at the grain-filling stage, where it significantly decreased.

### 3.2 Microbial community diversity

**Figure 2:**
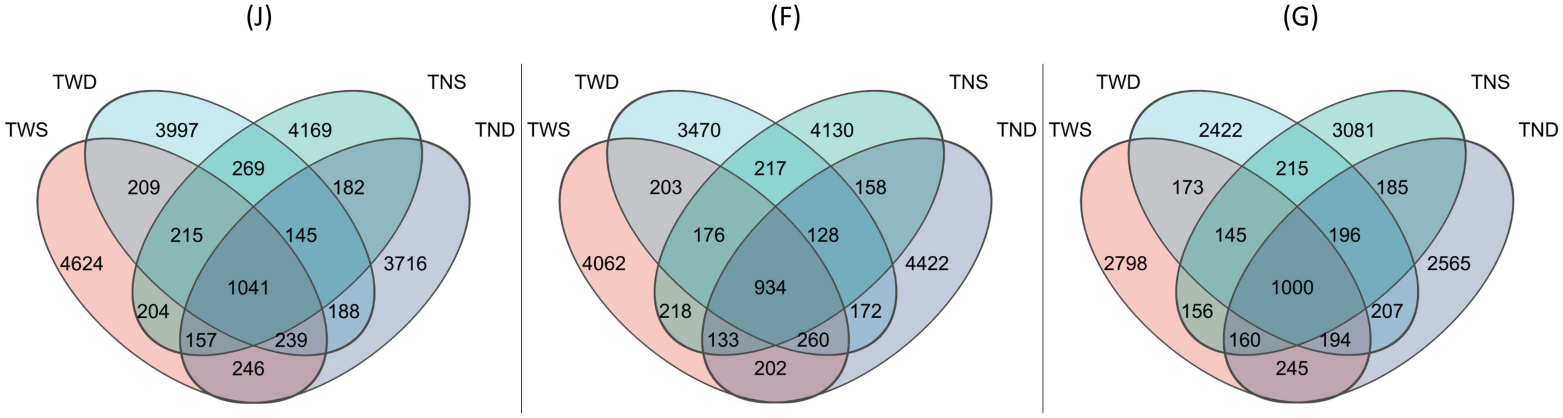
Venn diagram showing the abundance of Amplicon Sequence Variant (ASV) under different climatic conditions. TWS warming + full irrigation; TWD warming + deficit irrigation; TNS ambient temperature + full irrigation; TND ambient temperature + deficit irrigation; J jointing stage; F flowering stage; G grain filling stage.

The ace, Chao, sobs and shannon diversity indices were used to assess the impact of different climatic stresses on the alpha diversity of the soil bacterial community. The ANOVA test showed that the different climatic conditions and growth stages of winter wheat had various and significant influences on the diversity of the community (p<0.05) (**Tables 2**, **3**). Overall, the diversity indices and the number of amplicon sequence variants (ASVs) decreased as the wheat growth stage progressed (**Table 3**; **Figure 2**). At the jointing stage, diversity indices were higher under TWS and lower under TND, while at the flowering stage, indices were high under both TWS and TND (**Table 2**). Compared with the control treatment, TWD had no significant influence on the indices at any stage (**Table 2**). No significant difference was observed between the diversity indices under the different treatments at the grain-filling stage.

**Table 2:** Alpha diversity index of the soil bacterial community under different treatments.

Treatments		ace	Chao	sobs	Shannon
J	TWS	2982.15 a	2919.67 a	2895.67 a	7.145 a
	TWD	2718.12 b	2671.15 ab	2654.00 ab	7.036 ab
	TNS	2697.38 b	2661.14 ab	2648.00 ab	7.116 a
	TND	2468.38 b	2445.08 b	2436.33 b	6.850 b
	*p-value*	*0.003*	*0.016*	*0.015*	*0.011*
F	TWS	2616.65 a	2578.64 a	2572.33 a	7.074 a
	TWD	2401.19 b	2369.81 b	2360.00 b	6.963 a
	TNS	2438.36 b	2426.69 b	2424.00 b	7.021 a
	TND	2610.33 a	2588.41 a	2578.33 a	7.004 a
	*p-value*	*0.003*	*0.001*	*0.001*	*0.47*
G	TWS	2109.67 a	2097.20 a	2092.33 a	6.902 a
	TWD	1995.54 a	1990.34 a	1989.67 a	6.861 a
	TNS	2156.44 a	2144.98 a	2143.33 a	6.943 a
	TND	2098.40 a	2078.77 a	2071.33 a	6.844 a
	*p-value*	*0.33*	*0.35*	*0.35*	*0.63*

TWS warming + full irrigation; TWD warming + deficit irrigation; TNS ambient temperature + full irrigation; TND ambient temperature and deficit irrigation; J jointing stage; F flowering stage; G grain filling stage. Means followed by the same letter within the same column for each growth stage are not significantly different at α = 0.05, according to Tukey’s test.

**Table 3:** Alpha diversity index of the soil bacterial community at different wheat growth stages.

Stage	ace	Chao	sobs	Shannon
J	2716.51 a	2674.26 a	2658.50 a	7.036 a
F	2516.63 b	2490.89 b	2483.67 b	7.015 a
G	2090.01 c	2077.82 c	2074.17 c	6.887 b
*p-value*	*0.001*	*0.001*	*0.001*	*0.004*

TWS warming + full irrigation; TWD warming + deficit irrigation; TNS ambient temperature + full irrigation; TND ambient temperature and deficit irrigation; J jointing stage; F flowering stage; G grain filling stage. Means followed by the same letter within the same column are not significantly different at α = 0.05, according to Tukey’s test.

**Figure 3:**
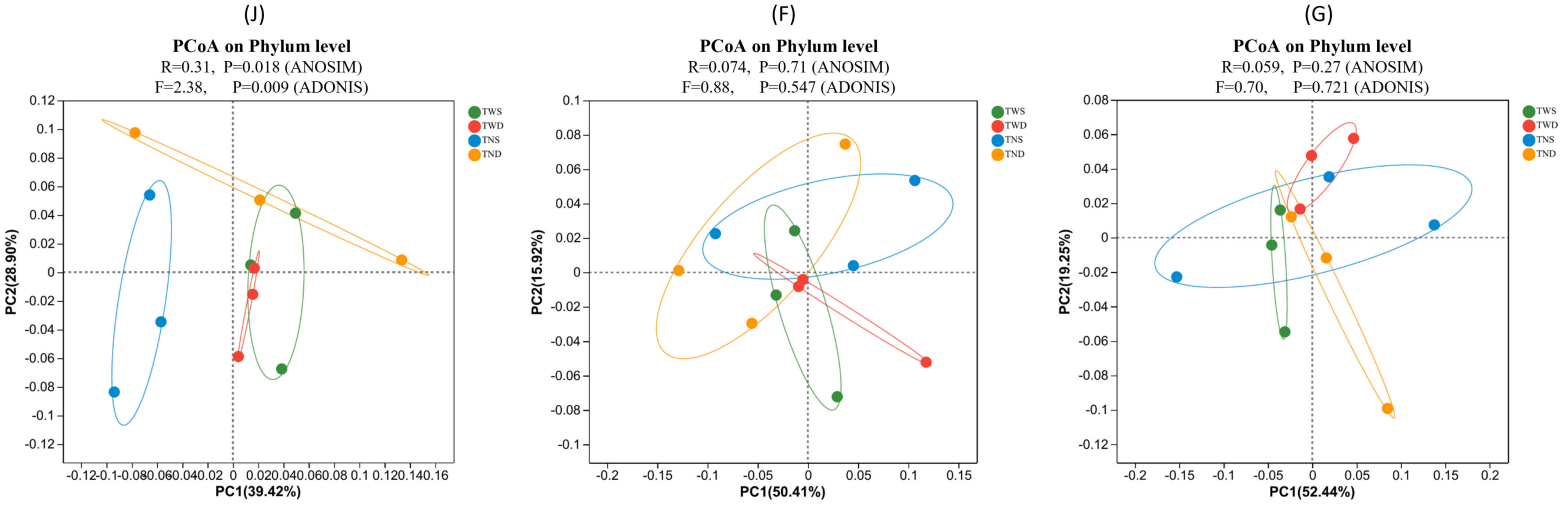
Principal coordinate analysis plot based on Bray–Curtis distance under the different climatic conditions. Each circle represents the bacterial community under one treatment. The closer the circles are, the greater the similarity between the bacterial communities they represent. TWS warming + full irrigation; TWD warming + deficit irrigation; TNS ambient temperature + full irrigation; TND ambient temperature + deficit irrigation; J jointing stage; F flowering stage; G grain filling stage. R shows the degree of difference between groups, and P is the significance of the R-value at α = 0.05.

Bray-Curtis distance analysis and ANOSIM were used to assess the similarity between the bacterial communities under different climatic conditions (**Figure 3**). ANOSIM test revealed a significant difference (p=0.018) between the bacterial communities under the different treatments at the jointing stage. In contrast, there was no significant difference at the other stages. At the jointing stage, the bacterial community under the control treatment was distant from the communities under the other treatments, and the communities under the treatments with warming (TWS and TWD) were closer to each other.

### 3.3 Abundance of microbial community

**Figure 4:**
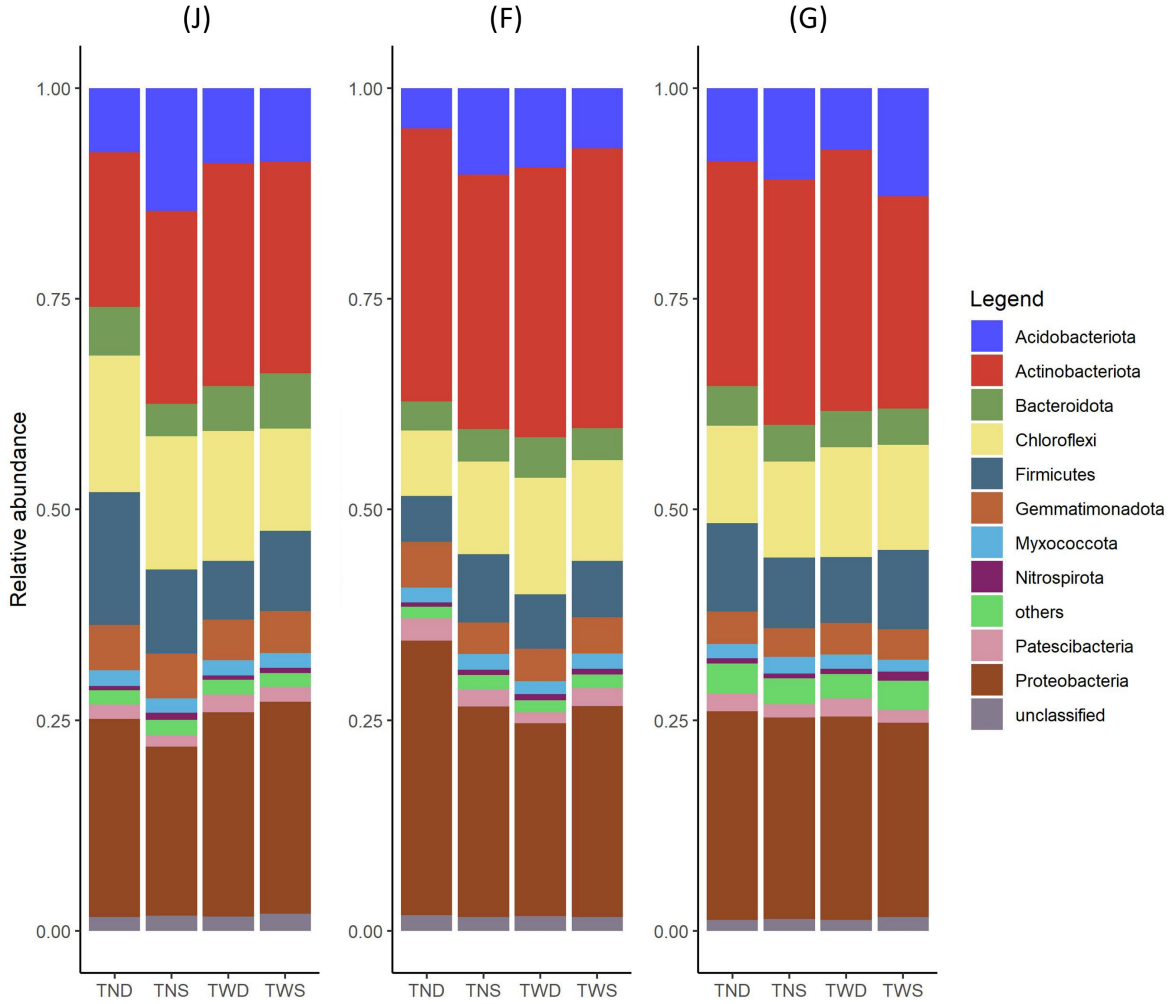
Relative abundance of soil bacteria communities at the phylum level under the different climatic conditions. Relative abundance was calculated by averaging the abundances of replicate samples. TWS warming + full irrigation; TWD warming + deficit irrigation; TNS ambient temperature + full irrigation; TND ambient temperature + deficit irrigation; J jointing stage; F flowering stage; G grain filling stage.

*Proteobacteria* (20-32%), *Actinobacteria* (17-32%), *Chroroflexi* (7-16%), *Firmicutes* (4-15%), and *Acidobacteria* (5-14%) were the five most dominant phyla under the different climatic conditions and at different stages of wheat growth. They represented about 80% of all the microbial taxa (**Figure 4**). The abundance of the bacterial community under TWS was relatively identical to that under TWD at the jointing stage but differed at the other growth stages. Compared with the other treatments, the abundance of *Acidobacteria* under TNS was greater at the jointing stage and lower at the other stages. A decrease in *Chloroflexi* and *Firmicutes* and an increase in *Actinobacteria* were observed under TND at the flowering and grain-filling stages compared with the jointing stage (**Figure 4**).

### 3.4 Co-occurrence network of the microbial community

**Figure 5:**
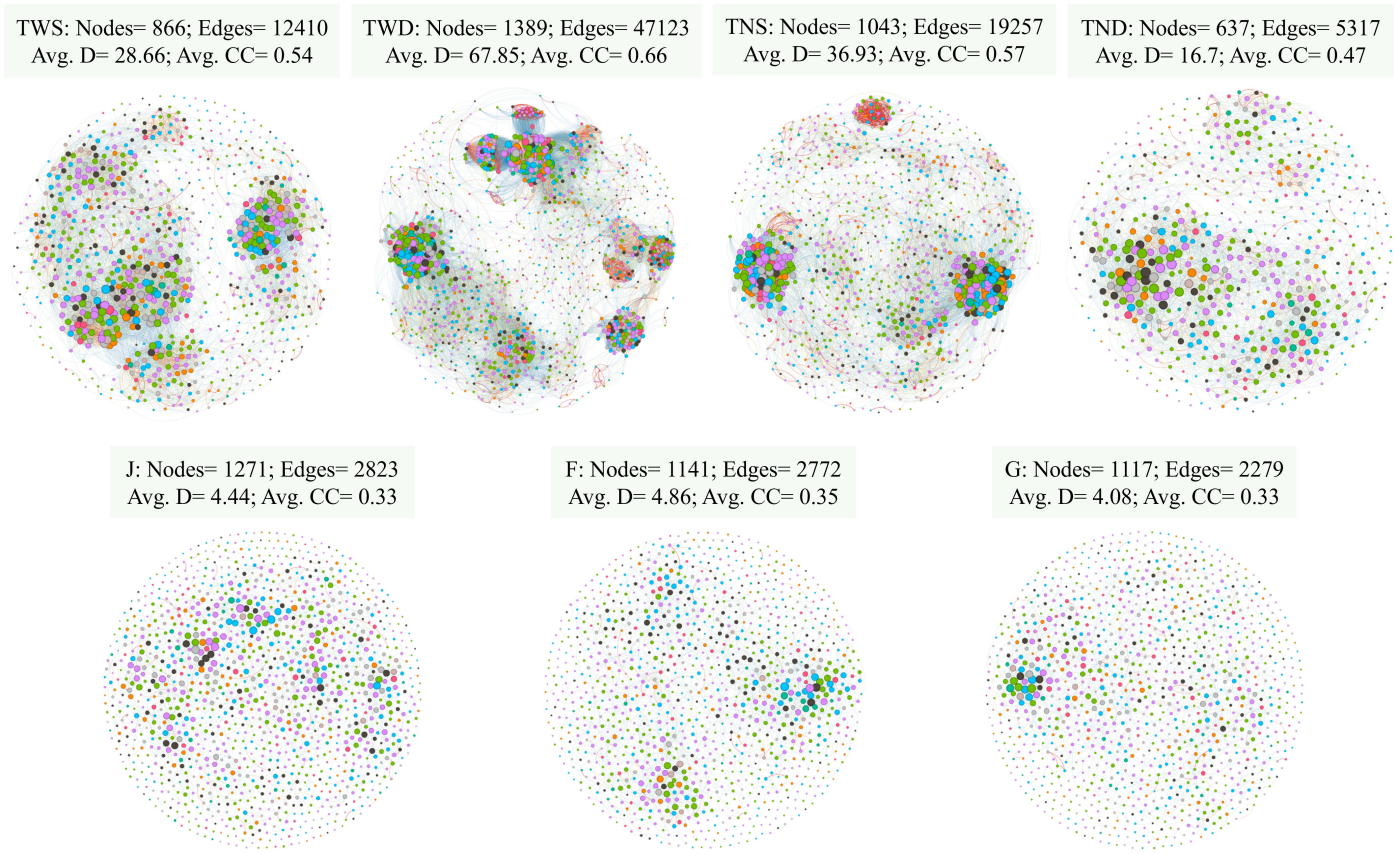
The bacterial co-occurrence networks for different treatments. Each node corresponds to an ASV, and edges between nodes correspond to either positive (red) or negative (blue) correlations. ASVs belonging to different microbial phyla have different color codes. TWS warming + full irrigation; TWD warming + deficit irrigation; TNS ambient temperature + full irrigation; TND ambient temperature + deficit irrigation; J jointing stage; F flowering stage; G grain filling stage; Avg. D average degree; Avg.CC average clustering coefficient.

The co-occurrence network of the soil bacterial community under each treatment is presented in **Figure 5**. Compared with the control treatment, the network feature values such as nodes, edges, average degree (Avg. D) and average clustering coefficient (Avg.CC) were higher under TWD and lower under TNS. These values under TWS were relatively close to those under the control treatment. These network features were almost identical at the different growth stages of the winter wheat.

### 3.5 Relationship between soil properties and the bacterial community

**Figure 6:**
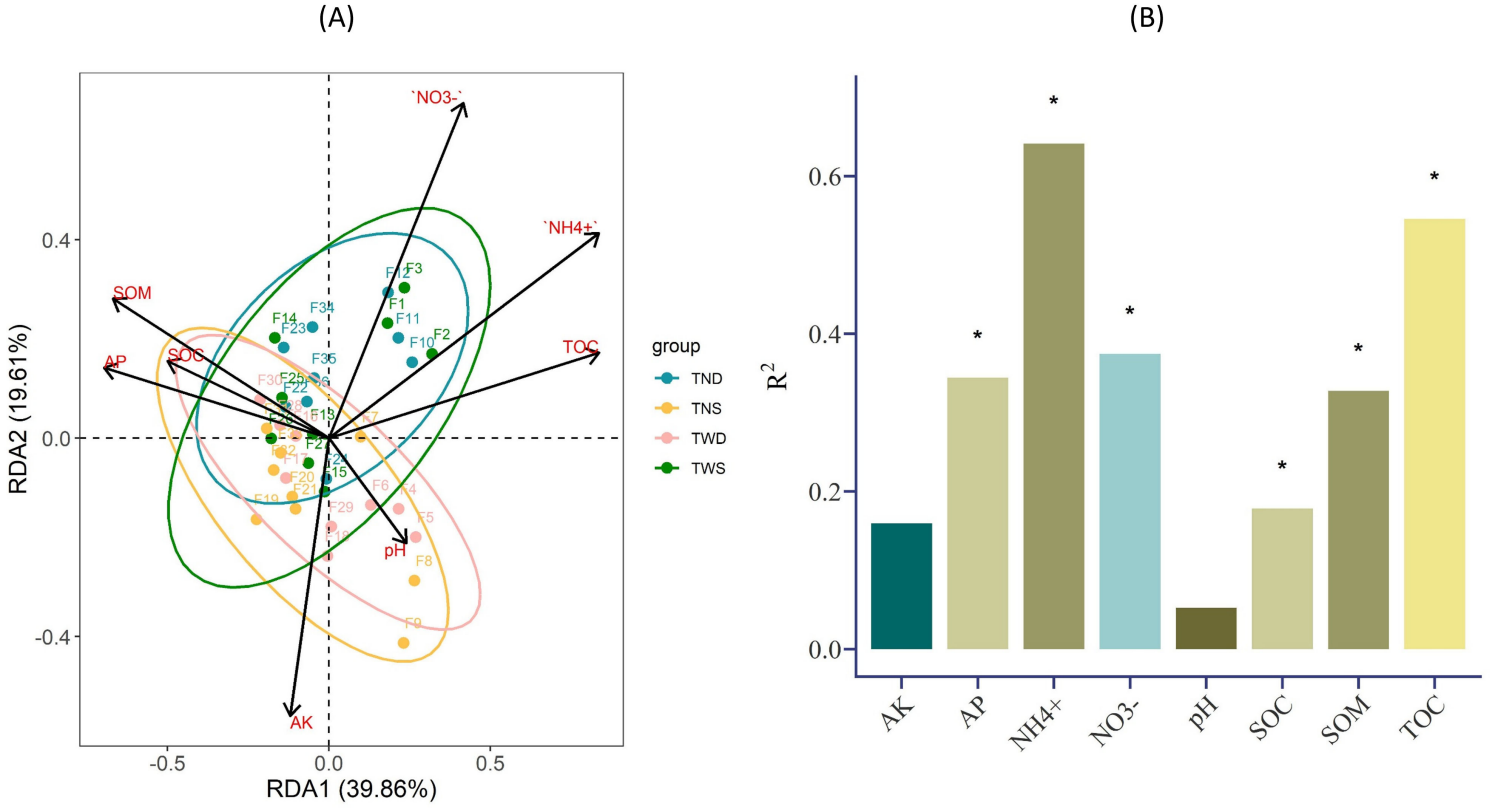
**(A)** Redundancy analysis (RDA) results of soil bacterial diversity under different climatic conditions, **(B)** Bar plot showing the level of significance of the correlation between the abundance of the soil bacterial community and environmental parameters. TWS warming + full irrigation; TWD warming + deficit irrigation; TNS ambient temperature + full irrigation; TND ambient temperature + deficit irrigation. The sign * shows the significant difference at α =0.05. NH4+ ammonium; NO3− nitrate; AP available phosphorous; AK available potassium; pH soil acidity; SOC soil organic carbon; SOM soil organic matter; TOC total organic carbon of root exudation.

**Figure 7:**
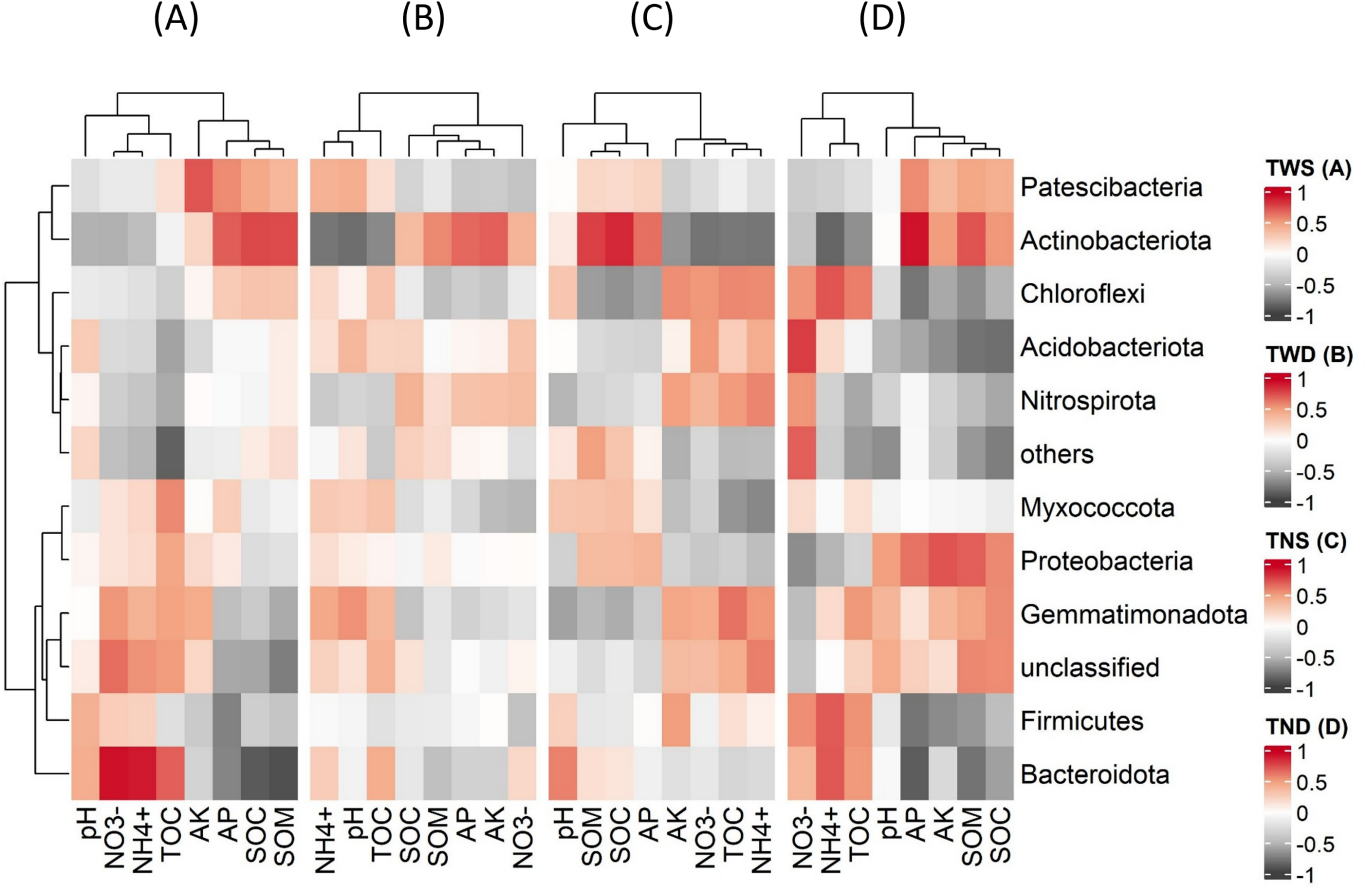
Pearson’s correlation between environmental parameters and bacterial abundance at the phylum level. TWS warming + full irrigation **(A)**; TWD warming + deficit irrigation **(B)**; TNS ambient temperature + full irrigation **(C)**; TND ambient temperature + deficit irrigation **(D)**. NH4+ ammonium; NO3− nitrate; AP available phosphorous; AK available potassium; pH soil acidity; SOC soil organic carbon; SOM soil organic matter; TOC total organic carbon of root exudation.

The soil chemical parameters and the TOC of root exudates were used to explain the variation in the abundance of the different bacterial phyla under different climatic conditions. **Figure 6B** shows that most of these parameters interacted significantly with the abundance of the soil bacterial community (p<0.05). According to **Figure 6A**, the bacterial community under TND and TWS interacted identically with NH_4_^+^, NO_3_^-^, and TOC, while that under TNS and TWD interacted identically with SOC, pH, and AP. Overall, the correlation between bacterial phyla and environmental parameters was weak under TWD but strong under TND (**Figure 7**).

Under TND, *Chloroflexi*, *Firmicutes*, and *Bacteroidota* were positively correlated with NH_4_^+^, NO_3_^-^, and TOC and negatively correlated with AP, AK, and SOC, while the opposite trend was observed with *Actinobacteria* and *Proteobacteria*. Unlike *Actinobacteria*, *Bacteroidota* was positively correlated with NH_4_^+^, NO_3_^-^, and TOC and negatively correlated with AP and SOC under TWS.

**Figure 8:**
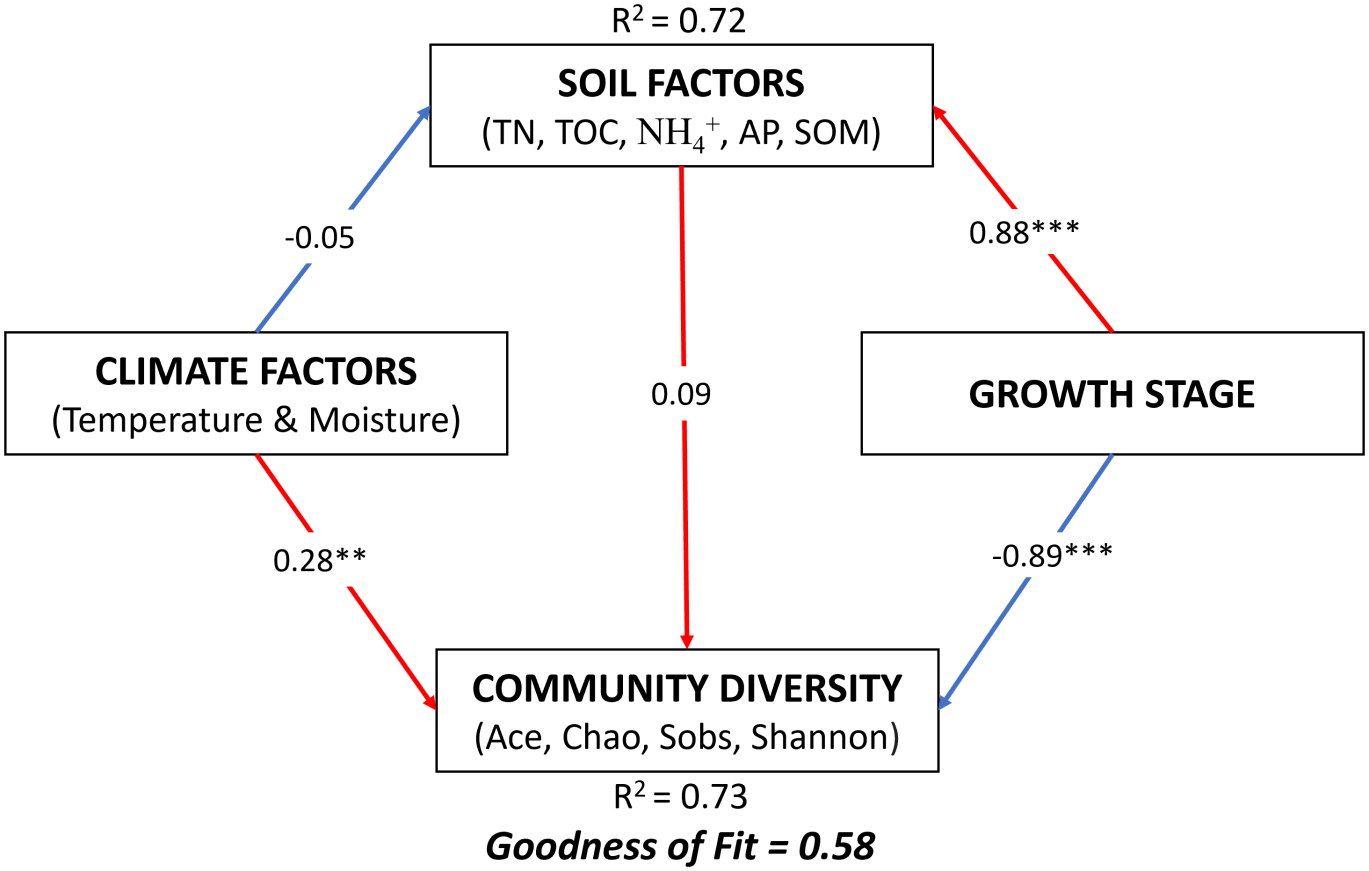
Partial least squares (PLS) illustrating the effect of soil, climatic conditions, and winter wheat growth stages on soil microbial community diversity. Numbers at arrows are indicative of the path coefficients. Red arrows represent positive relationships, whereas blue arrows represent negative relationships. Significance levels for each predictor are **p < 0.01, ***p < 0.001. TN total nitrogen; TOC total organic carbon of root exudation; NH4+ ammonium; AP available phosphorous; SOM soil organic matter.

The PLS analysis revealed the direct influences of climate, soil, and growth stages on the diversity of the soil bacterial community (**Figure 8**). According to this analysis, the growth stage exerted a significant influence on the soil (0.88) and community diversity (-0.89). The climate also directly influenced community diversity (0.28) and soil (-0.05), but its effect on the growth stage was indirect.

## 4 Discussion

### 4.1 Effects of different climatic stresses on soil nitrogen availability

Soil supports crops and is responsible for their defense and nutrition. Studies have shown that climate change affects the physico-chemical properties of the soil by altering the balance of the various reactions within it (Mondal, 2021; Pareek, 2017). This has repercussions on the availability of nutrients in the soil and the ability of plants to absorb them (Elbasiouny et al., 2022). The amounts of NH_4_^+^ and NO_3_^-^ were high at the jointing and flowering stages and low at the grain-filling stage under warming combined with a full irrigation treatment (**Table 1**). This would be due to an acceleration of mineralization and nitrogen uptake. Previous studies have reported similar results (Kaštovská et al., 2022; Dai et al., 2020; Liu et al., 2017). Warming accelerates the decomposition of nutrients in the soil (Dai et al., 2020) as well as their absorption by plants (Qiao et al., 2016) through an increase in evapotranspiration (Sadok et al., 2021). In addition, these climatic conditions induce significant nitrogen losses in the form of N_2_O (Medinets et al., 2021; Ma et al., 2022). The availability of high quantities of nitrogen at the early stage of growth would, therefore, be due to an acceleration of mineralization activities, and the low amounts observed at the advanced stages would be due to an acceleration of their uptake by the plants and an increase in the emission of NO_2_ into the atmosphere.

Contrary to the conditions of warming combined with full irrigation, the quantities of NH_4_^+^ and NO_3_^-^ under ambient temperature combined with deficit irrigation were low at the jointing stage and high at the other stages (**Table 1**). This can be explained by a slowdown in nitrogen mineralization in the soil and crop uptake. Reduced soil moisture limits microbial activity and oxygen diffusion, thereby suppressing nitrification and altering denitrification pathways, which decreases net mineralization of organic nitrogen. Deng et al. (2021) reported that drought reduces soil nitrogen mineralization but increases the quantity of mineral nitrogen in the soil. Xie and Shan (2021) demonstrated that water stress significantly increases the amount of NO_3_^-^ in the soil while inhibiting soil N losses. Other studies have reported the reducing effects of drought (Bista et al., 2018) and drought combined with warming (Hussain et al., 2019) on nitrogen uptake by crops. The high amount of nitrogen in the soil at the advanced stages of wheat growth would, therefore, be the consequence of its accumulation due to reduced plant uptake.

### 4.2 Effect of different climatic stresses on the soil organic carbon content

Root exudates mediate communication between plants and their underground environment. The results of the present study revealed an increase in the TOC content of root exudates, as well as the quantity of soil SOC under warming combined with full irrigation at all growth stages (**Table 1**). This suggests that elevated temperature under favorable moisture promoted greater allocation of assimilates belowground, leading to an increase in soil organic carbon. Similar trends were reported by Wang et al. (2021) and Zhang et al. (2016). Indeed, high-temperature conditions combined with full irrigation favor increased nutrient uptake (Hu et al., 2018) and biomass production by the plant (Silveira and Thiébaut, 2017). This, in turn, increases photosynthetic intensity (Liang et al., 2013; Moore et al., 2021) and consequently, the excessive release of organic compounds into the soil. The increase in the amount of SOC under warming combined with the full irrigation observed in the present study would result from the excessive release of organic carbon into the soil by winter wheat.

The TOC content under deficit irrigation combined with both ambient temperature and warming was identical to that under the control treatment at the different stages except at the grain-filling stage, where a significant drop in TOC was observed under ambient temperature combined with deficit irrigation. A decrease in SOC accompanied this decrease in TOC (**Table 1**). This would be due to the reduction in photosynthetic activity caused by drought and, therefore, a progressive decrease in TOC secretion by plants (Liu et al., 2018; Siddique et al., 2016). These results agree with those reported in previous studies (Karlowsky et al., 2018; Sanaullah et al., 2012).

### 4.3 Diversity of the soil bacterial community under different climatic stress conditions

Different climatic conditions affect the diversity of the soil microbial community in different ways. Previous studies have attempted to describe the impacts of climate change on bacterial community diversity in winter wheat fields (Preece et al., 2019; Siebielec et al., 2020; Wang et al., 2020; Wu et al., 2022). However, few research has considered the different growth stages of wheat. The results of the present study revealed that the influence of different climatic stresses on the alpha and beta diversity of the soil bacterial community varies with the plant growth stage (**Table 3**; **Figures 2**, **3**). This result is in agreement with other previous studies that reported the impact of different plant growth stages on the diversity of the soil microbial community under different environmental conditions (Collavino et al., 2020; Fu et al., 2023b, Navarro-Noya et al., 2022). The alpha diversity and dissimilarity of the bacterial community were highest at the jointing stage under all treatments and decreased with the advancing wheat growth stage (**Table 3**, **Figure 3**). Other studies have reported similar results (Wang et al., 2023b; Chen et al., 2019). Studies on millet have also reported a decrease in the diversity of the soil bacterial community at advanced growth stages of plants (Tian et al., 2022). This result may be due to the variation in the composition of root secretions at different growth stages of the plant (**Table 1**) (Gransee and Wittenmayer, 2000; Zhao et al., 2021). Root exudates considerably influence the diversity of the soil bacterial community (Kozdrój and Van Elsas, 2000; Nannipieri et al., 2008). Moreover, the composition of these exudates depends on several factors, including environmental conditions (Wang et al., 2021; Yin et al., 2013a, 2013) and the growth stage of the plant (Hasibeder et al., 2015). Warming combined with full irrigation increases the alpha diversity of the soil bacterial community at all growth stages of wheat (**Table 2**). Similar results have been obtained by other researchers (Fang et al., 2021; Wang et al., 2020; Zhai et al., 2024). However, the combined effect of warming and drought on the community’s alpha diversity remains less well understood. The present study shows that the latter climatic condition has no significant impact on the diversity of the soil bacterial community compared with the control condition (**Table 2**). This can be explained by the fact that drought inhibits the positive effects of higher temperatures on the soil microbial community (Liu et al., 2022b). These results contradict the work of von Rein et al. (2016), who reported adverse effects, and that of Sheik et al. (2011), who reported positive effects of the combined impact of warming and drought on the diversity of the soil bacterial community. The combined effect of these two climatic anomalies on the alpha diversity of the soil bacterial community would, therefore, depend on the intensity of warming and drought.

Several studies agree that drought alters the alpha diversity of the soil microbial community (Preece et al., 2019; Siebielec et al., 2020; Zhai et al., 2024). In the present study, the alpha diversity increased at the flowering stage compared to the jointing and the grain filling stage. These show that the effect of deficit irrigation on the community’s alpha diversity varied according to wheat’s growth stage (**Table 2**). These results are supported by the work of Na et al. (2019), who showed the dependence between crop growth stage and the influence of drought on soil bacterial community diversity.

### 4.4 Abundance and composition of the bacterial community under different climatic stress conditions

The abundance of the bacterial community under warming combined with full irrigation was relatively similar to that under warming combined with deficit irrigation at the jointing stage and different at the other growth stages of winter wheat (**Figure 4**). Moreover, high similarity between the bacterial communities under these two treatments was observed at the jointing stage (**Figure 3**). This shows that at the jointing stage, the bacterial community was more reactive to rising temperatures than to water deficit. Previous work has reported a variation in the abundance and composition of the soil bacterial community according to the growth stage of the plant (Houlden et al., 2008; Xiong et al., 2021). Wang et al. (2023a) reported significant influences of warming on the abundance of different phyla of the soil bacterial community at wheat’s tillering and jointing stage. Our findings highlight the jointing stage as the most sensitive period to warming, both in terms of soil nitrogen dynamics and rhizosphere microbial responses. This heightened sensitivity can be linked to various interconnected physiological and developmental processes because the jointing stage represents the transition from the vegetative to the reproductive stage.

The abundance of *Chloroflexi* and *Firmicutes* decreased while that of *Actinobacteria* increased under deficit irrigation at the flowering and grain-filling stages compared with the jointing stage (**Figure 4**). Previous studies have also reported a decrease in the abundance of *Firmicutes* (Kpalari et al., 2023; Zhang et al., 2019) and *Chloroflexi* (Dai et al., 2019; Kang et al., 2022; Preece et al., 2019) and an increase in *Actinobacteria* (Kang et al., 2022; Su et al., 2020; Xue et al., 2018) under drought conditions. Other studies have also demonstrated the contribution of bacteria of the genus *Bacillus*, *Paenibacillus*, and *Effusibacillus* (*Firmicutes*) in improving plant resistance to drought (Santander et al., 2024; Arunrat et al., 2025b). Moreover, *Actinobacteria* is one of the bacterial phyla containing the most species that promote plant growth (Hamedi and Mohammadipanah, 2015; Palaniyandi et al., 2013; Sathya et al., 2017). Thus, the phyla *Firmicutes*, *Chloroflexia*, and *Actinobacteria* would play diverse but essential roles in soil under drought conditions.

### 4.5 Effects of different climatic stresses on co-occurrence network of bacterial community

The co-occurrence patterns of bacterial communities were more influenced by climatic conditions than the growth stages of winter wheat (**Figure 5**). Warming combined with deficit irrigation considerably increased the network feature values, including nodes and edges, whereas this increase was more moderate under warming combined with full irrigation. Research by Yuan et al. (2021) revealed an improvement in the stability and complexity of the microbial network under warming, but the water content of the soil used for the experiment was not specified. Other studies have also reported similar results (Yu et al., 2021b; Liu et al., 2024a). The results of the present study show that the improvement in the complexity of the bacterial network under warming depends on the water content of the soil.

In contrast to warming combined with deficit irrigation, the ambient temperature combined with deficit irrigation reduced the network feature values. This is in accordance with the work of De Vries et al. (2018), who reported a decrease in bacterial network stability under drought conditions. According to Zhang et al. (2024), drought leads to a simplification of the microbial network, which in turn promotes a reduction in its stability and soil functionality.

### 4.6 Influence of different climatic stresses on the relationships between the bacterial community and environmental parameters

*Chloroflexi*, *Firmicutes*, and *Bacteroidota*, unlike *Actinobacteria* and *Proteobacteria*, were positively correlated with NO_3_^-^, NH_4_^+^, TOC, and negatively correlated with AP, AK, SOM, and SOC under ambient temperature combined with deficit irrigation (**Figure 7**). This highlights the involvement of these different bacterial phyla in the transformation of macronutrients and organic matter in the soil under drought conditions. Several studies have reported positive relationships between soil organic carbon and bacteria belonging to the *Actinobacteria* and *Proteobacteria* phyla (Woolet and Whitman, 2020; Yang et al., 2021b; Yu et al., 2021a). Other studies have also shown the contributions of these two bacterial phyla to the mineralization of soil phosphorus (Soumare et al., 2021; Wei et al., 2019; Zhang et al., 2021). Xiao et al. (2022) and Niu et al. (2020) reported positive correlations between *Chloroflexi* and nitrogen mineralization in the soil. Research into biofertilization has also shown that Bacillus-based fertilizers (*Firmicutes*) reduce nitrogen mineralization in the soil and their loss in the form of nitrous oxide (Sun et al., 2020a, 2020). These previous studies confirm the positive correlations observed between *Actinobacteria*, *Proteobacteria*, and soil organic carbon and available phosphorus and between *Firmicutes*, *Actinobacteria*, *Chloroflexi*, and available forms of soil nitrogen. Furthermore, the results of this study show that these different bacterial phyla are the biological agents most involved in the transformation of soil nutrients under drought conditions.

*Bacteroidota* was negatively correlated with AP and SOC, and positively correlated with NO_3_^-^, NH_4_^+^, and TOC under warming combined with a full irrigation regime and under ambient temperature combined with deficit irrigation (**Figure 7**). Previous work has shown the role of *Bacteroidota* in mineralizing soil organic matter (Cui et al., 2023; Lan et al., 2022). Other research has also reported positive correlations between *Bacteroidota*, *Firmicutes*, and soil nitrogen mineralization (Li et al., 2023c; Sun et al., 2020b). Nevertheless, more studies are needed to better explain the behavior of these bacterial phyla under different climatic conditions.

PLS analysis showed that different growth stages had more influence on the alpha diversity of the bacterial community than climatic conditions and soil parameters (**Figure 8**). Other previous studies have also reported the impact of growth stage on soil microbial community diversity (Guo et al., 2020; Wang et al., 2016). These results reveal the need to consider the crop growth stages when studying factors affecting the microbial community in the crop rhizosphere.

## 5 Conclusion

This study evaluated the response of the soil bacterial community to different climatic stresses across different growth stages of winter wheat. These results suggest that the sensitivity of wheat’s rhizosphere bacterial community to the impact of different climatic stresses is not identical at all growth stages and that this community is more sensitive at the jointing stage than at other stages. In addition, warming combined with full irrigation, unlike drought, increases the abundance and diversity of the community, while the complexity of the community is improved by warming combined with deficit irrigation. Structural equation modeling have also revealed the importance of considering different crop growth stages when studying factors affecting the rhizosphere bacterial community. Future reseach involving other levels of warming and drought is needed to acquire all the knowledge needed to understand the impact of these two climatic phenomena on soil bacteria.

## Data Availability

The original contributions presented in the study are publicly available. This data can be found here: https://www.ncbi.nlm.nih.gov/bioproject/PRJNA1378891.

## References

[B1] Arnell N. W. Lowe J. A. Challinor A. J. Osborn T. J. (2019). Global and regional impacts of climate change at different levels of global temperature increase. Clim. Change 155, 377–391. doi: 10.1007/s10584-019-02464-z

[B2] Arunrat N. Sereenonchai S. Uttarotai T. (2025a). Effects of soil texture on microbial community composition and abundance under alternate wetting and drying in paddy soils of central Thailand. Sci. Rep. 15, 24155. doi: 10.1038/s41598-025-09843-w, PMID: 40619541 PMC12230117

[B3] Arunrat N. Uttarotai T. Mhuantong W. Kongsurakan P. Sereenonchai S. Hatano R. (2025b). Soil bacterial communities in a 10-year fallow rotational shifting cultivation field and an 85-year-old terraced paddy field in Northern Thailand. Environ. Sci. Europe. 37, 95. doi: 10.1186/s12302-025-01143-4

[B4] Bista D. R. Heckathorn S. A. Jayawardena D. M. Mishra S. Boldt J. K. (2018). Effects of drought on nutrient uptake and the levels of nutrient-uptake proteins in roots of drought-sensitive and-tolerant grasses. Plants 7, 28. doi: 10.3390/plants7020028, PMID: 29601475 PMC6027393

[B5] Bogati K. Walczak M. (2022). The impact of drought stress on soil microbial community, enzyme activities and plants. Agronomy 12, 189. doi: 10.3390/agronomy12010189

[B6] Bu X. Gu X. Zhou X. Zhang M. Guo Z. Zhang J. . (2018). Extreme drought slightly decreased soil labile organic C and N contents and altered microbial community structure in a subtropical evergreen forest. For. Ecol. Manage. 429, 18–27. doi: 10.1016/j.foreco.2018.06.036

[B7] Canarini A. Schmidt H. Fuchslueger L. Martin V. Herbold C. W. Zezula D. . (2021). Ecological memory of recurrent drought modifies soil processes via changes in soil microbial community. Nat. Commun. 12, 5308. doi: 10.1038/s41467-021-25675-4, PMID: 34489463 PMC8421443

[B8] Chandio A. A. Dash D. P. Nathaniel S. P. Sargani G. R. Jiang Y. (2023). Mitigation pathways towards climate change: Modelling the impact of climatological factors on wheat production in top six regions of China. Ecol. Model. 481, 110381. doi: 10.1016/j.ecolmodel.2023.110381

[B9] Che Z. Yu D. Chen K. Wang H. Yang Z. Liu F. . (2022). Effects of warming on microbial community characteristics in the soil surface layer of niaodao wetland in the qinghai lake basin. Sustainability 14, 15255. doi: 10.3390/su142215255

[B10] Chen S. Waghmode T. R. Sun R. Kuramae E. E. Hu C. Liu B. (2019). Root-associated microbiomes of wheat under the combined effect of plant development and nitrogen fertilization. Microbiome 7, 136. doi: 10.1186/s40168-019-0750-2, PMID: 31640813 PMC6806522

[B11] Chen S. Zhang T. Wang J. (2021). Warming but not straw application increased microbial biomass carbon and microbial biomass carbon/nitrogen: importance of soil moisture. Water. Air. Soil pollut. 232, 53. doi: 10.1007/s11270-021-05029-y

[B12] Collavino M. M. Cabrera E. R. Bruno C. Aguilar O. M. (2020). Effect of soil chemical fertilization on the diversity and composition of the tomato endophytic diazotrophic community at different stages of growth. Braz. J. Microbiol. 51, 1965–1975. doi: 10.1007/s42770-020-00373-3, PMID: 32895888 PMC7688869

[B13] Cui H. Chen P. He C. Jiang Z. Lan R. Yang J. (2023). Soil microbial community structure dynamics shape the rhizosphere priming effect patterns in the paddy soil. Sci. Total. Environ. 857, 159459. doi: 10.1016/j.scitotenv.2022.159459, PMID: 36252670

[B14] Dai A. Zhao T. Chen J. (2018). Climate change and drought: a precipitation and evaporation perspective. Curr. Climate Change Rep. 4, 301–312. doi: 10.1007/s40641-018-0101-6

[B15] Dai L. Zhang G. Yu Z. Ding H. Xu Y. Zhang Z. (2019). Effect of drought stress and developmental stages on microbial community structure and diversity in peanut rhizosphere soil. Int. J. Mol. Sci. 20, 2265. doi: 10.3390/ijms20092265, PMID: 31071918 PMC6540327

[B16] Dai Z. Yu M. Chen H. Zhao H. Huang Y. Su W. . (2020). Elevated temperature shifts soil N cycling from microbial immobilization to enhanced mineralization, nitrification and denitrification across global terrestrial ecosystems. Global Change Biol. 26, 5267–5276. doi: 10.1111/gcb.15211, PMID: 32614503

[B17] Deng L. Peng C. Kim D.-G. Li J. Liu Y. Hai X. . (2021). Drought effects on soil carbon and nitrogen dynamics in global natural ecosystems. Earth-Sci. Rev. 214, 103501. doi: 10.1016/j.earscirev.2020.103501

[B18] De Vries F. T. Griffiths R. I. Bailey M. Craig H. Girlanda M. Gweon H. S. . (2018). Soil bacterial networks are less stable under drought than fungal networks. Nat. Commun. 9, 3033. doi: 10.1038/s41467-018-05516-7, PMID: 30072764 PMC6072794

[B19] Diffenbaugh N. S. Swain D. L. Touma D. (2015). Anthropogenic warming has increased drought risk in California. Proc. Natl. Acad. Sci. 112, 3931–3936. doi: 10.1073/pnas.1422385112, PMID: 25733875 PMC4386330

[B20] Duan S. Zhang Y. Zheng S. (2022). Heterotrophic nitrifying bacteria in wastewater biological nitrogen removal systems: A review. Crit. Rev. Environ. Sci. Technol. 52, 2302–2338. doi: 10.1080/10643389.2021.1877976

[B21] Elbasiouny H. El-Ramady H. Elbehiry F. Rajput V. D. Minkina T. Mandzhieva S. (2022). Plant nutrition under climate change and soil carbon sequestration. Sustainability 14, 914. doi: 10.3390/su14020914

[B22] Fang J. Wei S. Shi G. Cheng Y. Zhang X. Zhang F. Lu Z. Zhao X. (2021). Potential effects of temperature levels on soil bacterial community structure. E3S Web Conf., 292, 01008 doi: 10.1051/e3sconf/202129201008

[B23] Fawzy S. Osman A. I. Doran J. Rooney D. W. (2020). Strategies for mitigation of climate change: a review. Environ. Chem. Lett. 18, 2069–2094. doi: 10.1007/s10311-020-01059-w

[B24] Feng P. Wang B. Liu D. L. Waters C. Xiao D. Shi L. . (2020). Dynamic wheat yield forecasts are improved by a hybrid approach using a biophysical model and machine learning technique. Agricultural and Forest Meteorology, 285-286, 107922. doi: 10.1016/j.agrformet.2020.107922

[B25] Fu F. Li J. Li Y. Chen W. Ding H. Xiao S. (2023). Simulating the effect of climate change on soil microbial community in an Abies georgei var. smithii forest. Front. Microbiol. 14, 1189859. doi: 10.3389/fmicb.2023.1189859, PMID: 37333631 PMC10272780

[B26] Gransee A. Wittenmayer L. (2000). Qualitative and quantitative analysis of water-soluble root exudates in relation to plant species and development. J. Plant Nutr. Soil Sci. 163, 381–385. doi: 10.1002/1522-2624(200008)163:4<381::AID-JPLN381>3.0.CO;2-7

[B27] Guo Z. Wan S. Hua K. Yin Y. Chu H. Wang D. . (2020). Fertilization regime has a greater effect on soil microbial community structure than crop rotation and growth stage in an agroecosystem. Appl. Soil Ecol. 149, 103510. doi: 10.1016/j.apsoil.2020.103510

[B28] Hamedi J. Mohammadipanah F. (2015). Biotechnological application and taxonomical distribution of plant growth promoting actinobacteria. J. Ind. Microbiol. Biotechnol. 42, 157–171. doi: 10.1007/s10295-014-1537-x, PMID: 25410828

[B29] Hashimoto K. (2019). “ Global temperature and atmospheric carbon dioxide concentration,” in Global Carbon Dioxide Recycling: For Global Sustainable Development by Renewable Energy. Ed. Hashimoto K. ( Springer Singapore, Singapore).

[B30] Hasibeder R. Fuchslueger L. Richter A. Bahn M. (2015). Summer drought alters carbon allocation to roots and root respiration in mountain grassland. New Phytol. 205, 1117–1127. doi: 10.1111/nph.13146, PMID: 25385284 PMC4303983

[B31] Hoegh-Guldberg O. Jacob D. Taylor M. Guillén Bolaños T. Bindi M. Brown S. . (2019). The human imperative of stabilizing global climate change at 1.5 C. Science 365, eaaw6974. doi: 10.1126/science.aaw6974, PMID: 31604209

[B32] Houlden A. Timms-Wilson T. M. Day M. J. Bailey M. J. (2008). Influence of plant developmental stage on microbial community structure and activity in the rhizosphere of three field crops. FEMS Microbiol. Ecol. 65 2, 193–201. doi: 10.1111/j.1574-6941.2008.00535.x, PMID: 18616582

[B33] Hu C. Tian Z. Gu S. Guo H. Fan Y. Abid M. . (2018). Winter and spring night-warming improve root extension and soil nitrogen supply to increase nitrogen uptake and utilization of winter wheat (Triticum aestivum L.). Eur. J. Agron. 96, 96–107. doi: 10.1016/j.eja.2018.03.008

[B34] Hussain H. A. Men S. Hussain S. Chen Y. Ali S. Zhang S. . (2019). Interactive effects of drought and heat stresses on morpho-physiological attributes, yield, nutrient uptake and oxidative status in maize hybrids. Sci. Rep. 9, 3890. doi: 10.1038/s41598-019-40362-7, PMID: 30846745 PMC6405865

[B35] Kang E. Li Y. Zhang X. Yan Z. Zhang W. Zhang K. . (2022). Extreme drought decreases soil heterotrophic respiration but not methane flux by modifying the abundance of soil microbial functional groups in alpine peatland. Catena 212, 106043. doi: 10.1016/j.catena.2022.106043

[B36] Karlowsky S. Augusti A. Ingrisch J. Akanda M. K. U. Bahn M. Gleixner G. (2018). Drought-induced accumulation of root exudates supports post-drought recovery of microbes in mountain grassland. Front. Plant Sci. 9, 1593. doi: 10.3389/fpls.2018.01593, PMID: 30464767 PMC6234839

[B37] Karst J. Gaster J. Wiley E. Landhäusser S. M. (2017). Stress differentially causes roots of tree seedlings to exude carbon. Tree Physiol. 37, 154–164. doi: 10.1093/treephys/tpw090, PMID: 27744381

[B38] Kaštovská E. Choma M. Čapek P. Kaňa J. Tahovská K. Kopáček J. (2022). Soil warming during winter period enhanced soil N and P availability and leaching in alpine grasslands: A transplant study. PloS One 17, e0272143. doi: 10.1371/journal.pone.0272143, PMID: 35917373 PMC9345486

[B39] Khan M. Z. Maron P.-A. Dequiedt S. Rumpel C. Chabbi A. (2025). How does warming affect microbial communities in whole-soil under contrasting management? J. Soil Sci. Plant Nutr. doi: 10.1007/s42729-025-02598-3

[B40] Kome G. K. Enang R. K. Yerima B. P. K. (2018). Knowledge and management of soil fertility by farmers in western Cameroon. Geoderma. Reg. 13, 43–51. doi: 10.1016/j.geodrs.2018.02.001

[B41] Kozdrój J. Van Elsas J. D. (2000). Response of the bacterial community to root exudates in soil polluted with heavy metals assessed by molecular and cultural approaches. Soil Biol. Biochem. 32, 1405–1417. doi: 10.1016/S0038-0717(00)00058-4

[B42] Kpalari D. F. Mounkaila Hamani A. K. Hui C. Sogbedji J. M. Liu J. Le Y. . (2023). Soil bacterial community and greenhouse gas emissions as responded to the coupled application of nitrogen fertilizer and microbial decomposing inoculants in wheat (*Triticum aestivum L.*) seedling stage under different water regimes. Agronomy 13, 2950. doi: 10.3390/agronomy13122950

[B43] Kumar A. Verma J. P. (2019). “ The role of microbes to improve crop productivity and soil health,” in Ecological wisdom inspired restoration engineering, Singapore: Springer Singapore. 249–265.

[B44] Lan J. Wang S. Wang J. Qi X. Long Q. Huang M. (2022). The shift of soil bacterial community after afforestation influence soil organic carbon and aggregate stability in karst region. Front. Microbiol. 13, 901126. doi: 10.3389/fmicb.2022.901126, PMID: 35832811 PMC9271926

[B45] Leuschner C. Tückmantel T. Meier I. C. (2022). Temperature effects on root exudation in mature beech (Fagus sylvatica L.) forests along an elevational gradient. Plant Soil 481, 147–163. doi: 10.1007/s11104-022-05629-5

[B46] Li Q. Gao Y. Hamani A. K. M. Fu Y. Liu J. Wang H. . (2023a). Effects of warming and drought stress on the coupling of photosynthesis and transpiration in winter wheat (Triticum aestivum L.). Appl. Sci. 13, 2759. doi: 10.3390/app13052759

[B47] Li G. Kim S. Han S. H. Chang H. Du D. Son Y. (2018). Precipitation affects soil microbial and extracellular enzymatic responses to warming. Soil Biol. Biochem. 120, 212–221. doi: 10.1016/j.soilbio.2018.02.014

[B48] Li X. Wang A. Huang D. Qian H. Luo X. Chen W. . (2023c). Patterns and drivers of soil net nitrogen mineralization and its temperature sensitivity across eastern China. Plant Soil 485, 475–488. doi: 10.1007/s11104-022-05843-1

[B49] Li J. Xie T. Zhu H. Zhou J. Li C. Xiong W. . (2021). Alkaline phosphatase activity mediates soil organic phosphorus mineralization in a subalpine forest ecosystem. Geoderma 404, 115376. doi: 10.1016/j.geoderma.2021.115376

[B50] Li W. Yuan L. Lan X. Shi R. Chen D. Feng D. . (2023b). Effects of long-term warming on soil prokaryotic communities in shrub and alpine meadows on the eastern edge of the Qinghai-Tibetan Plateau. Appl. Soil Ecol. 188, 104871. doi: 10.1016/j.apsoil.2023.104871

[B51] Liang J. Xia J. Liu L. Wan S. (2013). Global patterns of the responses of leaf-level photosynthesis and respiration in terrestrial plants to experimental warming. J. Plant Ecol. 6, 437–447. doi: 10.1093/jpe/rtt003

[B52] Lindsey R. Dahlman L. (2020). Climate change: Global temperature. Clim. Gov. 16.

[B53] Liu J. Guo Y. Bai Y. Camberato J. Xue J. Zhang R. (2018). Effects of drought stress on the photosynthesis in maize. Russian J. Plant Physiol. 65, 849–856. doi: 10.1134/S1021443718060092

[B54] Liu W. Jiang Y. Su Y. Smoak J. M. Duan B. (2022a). Warming affects soil nitrogen mineralization via changes in root exudation and associated soil microbial communities in a subalpine tree species Abies fabri. J. Soil Sci. Plant Nutr., 1–10. doi: 10.1007/s42729-021-00657-z

[B55] Liu J. Si Z. Li S. Wu L. Zhang Y. Wu X. . (2024b). Effects of water and nitrogen rate on grain-filling characteristics under high-low seedbed cultivation in winter wheat. J. Integr. Agric. 23, 4018–4031. doi: 10.1016/j.jia.2023.12.002

[B56] Liu G. Sun J. Xie P. Guo C. Zhu K. Tian K. (2024a). Climate warming enhances microbial network complexity by increasing bacterial diversity and fungal interaction strength in litter decomposition. Sci. Total. Environ. 908, 168444. doi: 10.1016/j.scitotenv.2023.168444, PMID: 37949122

[B57] Liu Y. Tian H. Li J. Wang H. Liu S. Liu X. (2022b). Reduced precipitation neutralizes the positive impact of soil warming on soil microbial community in a temperate oak forest. Sci. Total. Environ. 806, 150957. doi: 10.1016/j.scitotenv.2021.150957, PMID: 34656582

[B58] Liu X. Yang Z. Lin C. Giardina C. P. Xiong D. Lin W. . (2017). Will nitrogen deposition mitigate warming-increased soil respiration in a young subtropical plantation? Agric. For. Meteorol. 246, 78–85. doi: 10.1016/j.agrformet.2017.06.010

[B59] Lyu D. Backer R. Robinson W. G. Smith D. L. (2019). Plant growth-promoting rhizobacteria for cannabis production: yield, cannabinoid profile and disease resistance. Front. Microbiol. 1761. doi: 10.3389/fmicb.2019.01761, PMID: 31456755 PMC6698789

[B60] Ma C. Liu Y. Wang J. Xue L. Hou P. Xue L. . (2022). Warming increase the N2O emissions from wheat fields but reduce the wheat yield in a rice-wheat rotation system. Agricult. Ecosyst. Environ. 337, 108064. doi: 10.1016/j.agee.2022.108064

[B61] Ma Z. L. Zhao W. Q. Liu M. Liu Q. (2019). Effects of warming on microbial biomass carbon and nitrogen in the rhizosphere and bulk soil in an alpine scrub ecosystem. Ying. Yong. Sheng. Tai. Xue. Bao=. J. Appl. Ecol. 30, 1893–1900. doi: 10.13287/j.1001-9332.201906.024, PMID: 31257761

[B62] Marengo J. A. Torres R. R. Alves L. M. (2017). Drought in Northeast Brazil—past, present, and future. Theor. Appl. Climatol. 129, 1189–1200. doi: 10.1007/s00704-016-1840-8

[B63] Masson-Delmotte V. Zhai P. Pörtner H. O. Roberts D. Skea J. Shukla P. R. . (2018). Global Warming of 1.5˚C. An IPCC Special Report on the Impacts of Global Warming of 1. 5 C above Pre-Industrial Levels and Related Global Greenhouse Gas Emission Pathways, in the Context of Strengthening the Global Response to the Threat of Climate Change, Sustainable Development, and Efforts to Eradicate Poverty. Sustainable Development, and Efforts to Eradicate Poverty, 616.

[B64] Medinets S. White S. Cowan N. Drewer J. Dick J. Jones M. . (2021). Impact of climate change on soil nitric oxide and nitrous oxide emissions from typical land uses in Scotland. Environ. Res. Lett. 16, 055035. doi: 10.1088/1748-9326/abf06e

[B65] Mekala S. Polepongu S. (2019). “ Impact of climate change on soil microbial community,” in Plant biotic interactions: State of the art, Cham: Springer International Publishing. 31–41.

[B66] Mondal S. (2021). “ Impact of climate change on soil fertility,” in Climate change and the microbiome: sustenance of the ecosphere, Cham: Springer International Publishing. 551–569.

[B67] Monteleone B. Borzí I. Arosio M. Cesarini L. Bonaccorso B. Martina M. (2023). Modelling the response of wheat yield to stage-specific water stress in the Po Plain. Agric. Water Manage. 287, 108444. doi: 10.1016/j.agwat.2023.108444

[B68] Moore C. E. Meacham-Hensold K. Lemonnier P. Slattery R. A. Benjamin C. Bernacchi C. J. . (2021). The effect of increasing temperature on crop photosynthesis: from enzymes to ecosystems. J. Exp. Bot. 72, 2822–2844. doi: 10.1093/jxb/erab090, PMID: 33619527 PMC8023210

[B69] Na X. Cao X. Ma C. Ma S. Xu P. Liu S. . (2019). Plant stage, not drought stress, determines the effect of cultivars on bacterial community diversity in the rhizosphere of broomcorn millet (Panicum miliaceum L.). Front. Microbiol. 10, 828. doi: 10.3389/fmicb.2019.00828, PMID: 31068914 PMC6491785

[B70] Nakayama M. Tateno R. (2018). Solar radiation strongly influences the quantity of forest tree root exudates. Trees 32, 871–879. doi: 10.1007/s00468-018-1685-0

[B71] Nannipieri P. Ascher J. Ceccherini M. T. Landi L. Pietramellara G. Renella G. . (2008). “ Effects of root exudates in microbial diversity and activity in rhizosphere soils,” in Molecular Mechanisms of Plant and Microbe Coexistence. Eds. Nautiyal C. S. Dion P. ( Springer Berlin Heidelberg, Berlin, Heidelberg).

[B72] Navarro-Noya Y. E. Chávez-Romero Y. Hereira-Pacheco S. De León Lorenzana A. S. Govaerts B. Verhulst N. . (2022). Bacterial communities in the rhizosphere at different growth stages of maize cultivated in soil under conventional and conservation agricultural practices. Microbiol. Spectr. 10, e01834–e01821. doi: 10.1128/spectrum.01834-21, PMID: 35254138 PMC9049951

[B73] Ning D. Qin A. Duan A. Xiao J. Zhang J. Liu Z. . (2019). Deficit irrigation combined with reduced N-fertilizer rate can mitigate the high nitrous oxide emissions from Chinese drip-fertigated maize field. Global Ecol. Conserv. 20, e00803. doi: 10.1016/j.gecco.2019.e00803

[B74] Niu Y. Zhang M. Bai S. H. Xu Z. Liu Y. Chen F. . (2020). Successive mineral nitrogen or phosphorus fertilization alone significantly altered bacterial community rather than bacterial biomass in plantation soil. Appl. Microbiol. Biotechnol. 104, 7213–7224. doi: 10.1007/s00253-020-10761-2, PMID: 32632477

[B75] Ochoa-Hueso R. Collins S. L. Delgado-Baquerizo M. Hamonts K. Pockman W. T. Sinsabaugh R. L. . (2018). Drought consistently alters the composition of soil fungal and bacterial communities in grasslands from two continents. Global Change Biol. 24, 2818–2827. doi: 10.1111/gcb.14113, PMID: 29505170

[B76] Palaniyandi S. A. Yang S. H. Zhang L. Suh J.-W. (2013). Effects of actinobacteria on plant disease suppression and growth promotion. Appl. Microbiol. Biotechnol. 97, 9621–9636. doi: 10.1007/s00253-013-5206-1, PMID: 24092003

[B77] Pareek N. (2017). Climate change impact on soils: adaptation and mitigation. MOJ. Ecol. Environ. Sci. 2, 136–139. doi: 10.15406/mojes.2017.02.00026

[B78] Phillips R. P. Erlitz Y. Bier R. Bernhardt E. S. (2008). New approach for capturing soluble root exudates in forest soils. Funct. Ecol. 22, 990–999. doi: 10.1111/j.1365-2435.2008.01495.x

[B79] Preece C. Verbruggen E. Liu L. Weedon J. T. Peñuelas J. (2019). Effects of past and current drought on the composition and diversity of soil microbial communities. Soil Biol. Biochem. 131, 28–39. doi: 10.1016/j.soilbio.2018.12.022

[B80] Qiao M. Zhang Z. Li Y. Xiao J. Yin H. Yue B. . (2016). Experimental warming effects on root nitrogen absorption and mycorrhizal infection in a subalpine coniferous forest. Scand. J. For. Res. 31, 347–354. doi: 10.1080/02827581.2015.1080295

[B81] Ramette A. (2007). Multivariate analyses in microbial ecology. FEMS Microbiol. Ecol. 62, 142–160. doi: 10.1111/j.1574-6941.2007.00375.x, PMID: 17892477 PMC2121141

[B82] Rashid M. Kamruzzaman M. Haque A. Krehenbrink M. (2019). “ Soil microbes for sustainable agriculture,” in Sustainable management of soil and environment, Cham: Springer International Publishing. 339–382.

[B83] Reimer M. Hartmann T. E. Oelofse M. Magid J. Bünemann E. K. Möller K. (2020). Reliance on biological nitrogen fixation depletes soil phosphorus and potassium reserves. Nutrient. Cycling. Agroecosyst. 118, 273–291. doi: 10.1007/s10705-020-10101-w

[B84] Sadok W. Lopez J. R. Smith K. P. (2021). Transpiration increases under high-temperature stress: Potential mechanisms, trade-offs and prospects for crop resilience in a warming world. Plant. Cell Environ. 44, 2102–2116. doi: 10.1111/pce.13970, PMID: 33278035

[B85] Sanaullah M. Chabbi A. Rumpel C. Kuzyakov Y. (2012). Carbon allocation in grassland communities under drought stress followed by 14C pulse labeling. Soil Biol. Biochem. 55, 132–139. doi: 10.1016/j.soilbio.2012.06.004

[B86] Santander C. González F. Pérez U. Ruiz A. Aroca R. Santos C. . (2024). Enhancing Water Status and Nutrient Uptake in Drought-Stressed Lettuce Plants (Lactuca sativa L.) via Inoculation with Different Bacillus spp. Isolated from the Atacama Desert. Plants 13, 158. doi: 10.3390/plants13020158, PMID: 38256712 PMC10818642

[B87] Sathya A. Vijayabharathi R. Gopalakrishnan S. (2017). Plant growth-promoting actinobacteria: a new strategy for enhancing sustainable production and protection of grain legumes. 3 Biotech. 7, 1–10. doi: 10.1007/s13205-017-0736-3, PMID: 28560641 PMC5449283

[B88] Schimel J. P. (2018). Life in dry soils: effects of drought on soil microbial communities and processes. Annu. Rev. Ecol. Evol. syst. 49, 409–432. doi: 10.1146/annurev-ecolsys-110617-062614

[B89] Schloter M. Nannipieri P. Sørensen S. J. Van Elsas J. D. (2018). Microbial indicators for soil quality. Biol. Fertil. Soils. 54, 1–10. doi: 10.1007/s00374-017-1248-3

[B90] Sheik C. S. Beasley W. H. Elshahed M. S. Zhou X. Luo Y. Krumholz L. R. (2011). Effect of warming and drought on grassland microbial communities. ISME. J. 5, 1692–1700. doi: 10.1038/ismej.2011.32, PMID: 21451582 PMC3176507

[B91] Shen Z. Yu B. Shao K. Gao G. Tang X. (2023). Warming reduces microeukaryotic diversity, network complexity and stability. Environ. Res. 238, 117235. doi: 10.1016/j.envres.2023.117235, PMID: 37775010

[B92] Shoukat M. R. Wang J. Habib-Ur-Rahman M. Hui X. Hoogenboom G. Yan H. (2024). Adaptation strategies for winter wheat production at farmer fields under a changing climate: Employing crop and multiple global climate models. Agric. Syst. 220, 104066. doi: 10.1016/j.agsy.2024.104066

[B93] Siddique Z. Jan S. Imadi S. R. Gul A. Ahmad P. (2016). Drought stress and photosynthesis in plants. Water Stress Crop Plants.: Sustain. Approach. 1, 1–11. doi: 10.1002/9781119054450.ch1

[B94] Siebielec S. Siebielec G. Klimkowicz-Pawlas A. Gałązka A. Grządziel J. Stuczyński T. (2020). Impact of water stress on microbial community and activity in sandy and loamy soils. Agronomy 10, 1429. doi: 10.3390/agronomy10091429

[B95] Silveira M. J. Thiébaut G. (2017). Impact of climate warming on plant growth varied according to the season. Limnologica 65, 4–9. doi: 10.1016/j.limno.2017.05.003

[B96] Soumare A. Boubekri K. Lyamlouli K. Hafidi M. Ouhdouch Y. Kouisni L. (2021). Efficacy of phosphate solubilizing Actinobacteria to improve rock phosphate agronomic effectiveness and plant growth promotion. Rhizosphere 17, 100284. doi: 10.1016/j.rhisph.2020.100284

[B97] Staszel K. Lasota J. Błońska E. (2022). Effect of drought on root exudates from Quercus petraea and enzymatic activity of soil. Sci. Rep. 12, 7635. doi: 10.1038/s41598-022-11754-z, PMID: 35538167 PMC9090927

[B98] Su X. Su X. Zhou G. Du Z. Yang S. Ni M. . (2020). Drought accelerated recalcitrant carbon loss by changing soil aggregation and microbial communities in a subtropical forest. Soil Biol. Biochem. 148, 107898. doi: 10.1016/j.soilbio.2020.107898

[B99] Sun B. Bai Z. Bao L. Xue L. Zhang S. Wei Y. . (2020a). Bacillus subtilis biofertilizer mitigating agricultural ammonia emission and shifting soil nitrogen cycling microbiomes. Environ. Int. 144, 105989. doi: 10.1016/j.envint.2020.105989, PMID: 32739514

[B100] Sun Y. Chen H. Y. Jin L. Wang C. Zhang R. Ruan H. . (2020c). Drought stress induced increase of fungi: bacteria ratio in a poplar plantation. Catena 193, 104607. doi: 10.1016/j.catena.2020.104607

[B101] Sun B. Gu L. Bao L. Zhang S. Wei Y. Bai Z. . (2020b). Application of biofertilizer containing Bacillus subtilis reduced the nitrogen loss in agricultural soil. Soil Biol. Biochem. 148, 107911. doi: 10.1016/j.soilbio.2020.107911

[B102] Sun H. Wang Y. Wang L. (2024). Impact of climate change on wheat production in China. Eur. J. Agron. 153, 127066. doi: 10.1016/j.eja.2023.127066

[B103] Tian L. Yu S. Zhang L. Dong K. Feng B. (2022). Mulching practices manipulate the microbial community diversity and network of root−associated compartments in the Loess Plateau. Soil Tillage. Res. 223, 105476. doi: 10.1016/j.still.2022.105476

[B104] Ulrich D. E. Clendinen C. S. Alongi F. Mueller R. C. Chu R. K. Toyoda J. . (2022). Root exudate composition reflects drought severity gradient in blue grama (Bouteloua gracilis). Sci. Rep. 12, 12581. doi: 10.1038/s41598-022-16408-8, PMID: 35869127 PMC9307599

[B105] Veach A. M. Zeglin L. H. (2020). Historical drought affects microbial population dynamics and activity during soil drying and re-wet. Microbial. Ecol. 79, 662–674. doi: 10.1007/s00248-019-01432-5, PMID: 31482287

[B106] Verma M. Mishra J. Arora N. K. (2019). Plant Growth-Promoting Rhizobacteria: Diversity and Applications. In: Environmental Biotechnology: For Sustainable Future. Singapore: Springer Singapore 129–173. doi: 10.1007/978-981-10-7284-0_6

[B107] Vicente-Serrano S. M. Quiring S. M. Pena-Gallardo M. Yuan S. Dominguez-Castro F. (2020). A review of environmental droughts: Increased risk under global warming? Earth-Sci. Rev. 201, 102953. doi: 10.1016/j.earscirev.2019.102953

[B108] Vocciante M. Grifoni M. Fusini D. Petruzzelli G. Franchi E. (2022). The role of plant growth-promoting rhizobacteria (PGPR) in mitigating plant’s environmental stresses. Appl. Sci. 12, 1231. doi: 10.3390/app12031231

[B109] Von Rein I. Gessler A. Premke K. Keitel C. Ulrich A. Kayler Z. E. (2016). Forest understory plant and soil microbial response to an experimentally induced drought and heat-pulse event: the importance of maintaining the continuum. Global Change Biol. 22, 2861–2874. doi: 10.1111/gcb.13270, PMID: 26946456

[B110] Walkley A. Black I. A. (1934). An examination of the Degtjareff method for determining soil organic matter, and a proposed modification of the chromic acid titration method. Soil Sci. 37, 29–38. doi: 10.1097/00010694-193401000-00003

[B111] Wang J. Chen S. Sun R. Liu B. Waghmode T. Hu C. (2023a). Spatial and temporal dynamics of the bacterial community under experimental warming in field-grown wheat. PeerJ 11, e15428. doi: 10.7717/peerj.15428, PMID: 37334112 PMC10276554

[B112] Wang Q. Chen L. Xu H. Ren K. Xu Z. Tang Y. . (2021). The effects of warming on root exudation and associated soil N transformation depend on soil nutrient availability. Rhizosphere 17, 100263. doi: 10.1016/j.rhisph.2020.100263

[B113] Wang X. Jiang D. Lang X. (2018). Climate change of 4 C global warming above pre-industrial levels. Adv. Atmospheric. Sci. 35, 757–770. doi: 10.1007/s00376-018-7160-4

[B114] Wang Y. Ma A. Chong G.-S. Xie F. Zhou H. Liu G. . (2020). Effect of simulated warming on microbial community in glacier forefield. Huan. Jing. Ke. Xue= Huanjing. Kexue. 41 6, 2918–2923. doi: 10.13227/j.hjkx.201911157, PMID: 32608809

[B115] Wang P. Nie J. Yang L. Zhao J. Wang X. Zhang Y. . (2023b). Plant growth stages covered the legacy effect of rotation systems on microbial community structure and function in wheat rhizosphere. Environ. Sci. pollut. Res. 30, 59632–59644. doi: 10.1007/s11356-023-26703-0, PMID: 37012567

[B116] Wang J. Xue C. Song Y. Wang L. Huang Q. Shen Q. (2016). Wheat and rice growth stages and fertilization regimes alter soil bacterial community structure, but not diversity. Front. Microbiol. 7. doi: 10.3389/fmicb.2016.01207, PMID: 27536292 PMC4971054

[B117] Wei X. Hu Y. Razavi B. S. Zhou J. Shen J. Nannipieri P. . (2019). Rare taxa of alkaline phosphomonoesterase-harboring microorganisms mediate soil phosphorus mineralization. Soil Biol. Biochem. 131, 62–70. doi: 10.1016/j.soilbio.2018.12.025

[B118] Woolet J. Whitman T. (2020). Pyrogenic organic matter effects on soil bacterial community composition. Soil Biol. Biochem. 141, 107678. doi: 10.1016/j.soilbio.2019.107678

[B119] Wu L. Zhang Y. Guo X. Ning D. Zhou X. Feng J. . (2022). Reduction of microbial diversity in grassland soil is driven by long-term climate warming. Nat. Microbiol. 7, 1054–1062. doi: 10.1038/s41564-022-01147-3, PMID: 35697795

[B120] Xiao R. Man X. Duan B. Cai T. Ge Z. Li X. . (2022). Changes in soil bacterial communities and nitrogen mineralization with understory vegetation in boreal larch forests. Soil Biol. Biochem. 166, 108572. doi: 10.1016/j.soilbio.2022.108572

[B121] Xie T. Shan L. (2021). Water stress and appropriate N management achieves profitable yields and less N loss on sandy soils. Arid. Land. Res. Manage. 35, 358–373. doi: 10.1080/15324982.2020.1868024

[B122] Xiong C. Singh B. K. He J.-Z. Han Y.-L. Li P.-P. Wan L.-H. . (2021). Plant developmental stage drives the differentiation in ecological role of the maize microbiome. Microbiome 9, 171. doi: 10.1186/s40168-021-01118-6, PMID: 34389047 PMC8364065

[B123] Xu L. Chen N. Zhang X. (2019). Global drought trends under 1.5 and 2 C warming. Int. J. Climatol. 39, 2375–2385. doi: 10.1002/joc.5958

[B124] Xu S. Geng W. Sayer E. J. Zhou G. Zhou P. Liu C. (2020). Soil microbial biomass and community responses to experimental precipitation change: A meta-analysis. Soil Ecol. Lett. 2, 93–103. doi: 10.1007/s42832-020-0033-7

[B125] Xu W. Yuan W. (2017). Responses of microbial biomass carbon and nitrogen to experimental warming: A meta-analysis. Soil Biol. Biochem. 115, 265–274. doi: 10.1016/j.soilbio.2017.08.033

[B126] Xue P.-P. Carrillo Y. Pino V. Minasny B. Mcbratney A. B. (2018). Soil properties drive microbial community structure in a large scale transect in South Eastern Australia. Sci. Rep. 8, 11725. doi: 10.1038/s41598-018-30005-8, PMID: 30082740 PMC6078944

[B127] Yang S. Jansen B. Absalah S. Kalbitz K. Castro F. O. C. Cammeraat E. L. (2022). Soil organic carbon content and mineralization controlled by the composition, origin and molecular diversity of organic matter: a study in tropical alpine grasslands. Soil Tillage. Res. 215, 105203. doi: 10.1016/j.still.2021.105203

[B128] Yang Y. Li T. Wang Y. Cheng H. Chang S. X. Liang C. . (2021a). Negative effects of multiple global change factors on soil microbial diversity. Soil Biol. Biochem. 156, 108229. doi: 10.1016/j.soilbio.2021.108229

[B129] Yang Y. Liu H. Dai Y. Tian H. Zhou W. Lv J. (2021b). Soil organic carbon transformation and dynamics of microorganisms under different organic amendments. Sci. Total. Environ. 750, 141719. doi: 10.1016/j.scitotenv.2020.141719, PMID: 32858285

[B130] Yin H. Li Y. Xiao J. Xu Z. Cheng X. Liu Q. (2013a). Enhanced root exudation stimulates soil nitrogen transformations in a subalpine coniferous forest under experimental warming. Global Change Biol. 19, 2158–2167. doi: 10.1111/gcb.12161, PMID: 23504744

[B131] Yin H. Xiao J. Li Y. Chen Z. Cheng X. Zhao C. . (2013b). Warming effects on root morphological and physiological traits: The potential consequences on soil C dynamics as altered root exudation. Agric. For. Meteorol. 180, 287–296. doi: 10.1016/j.agrformet.2013.06.016

[B132] You X. Wang S. Du L. Wu H. Wei Y. (2022). Effects of organic fertilization on functional microbial communities associated with greenhouse gas emissions in paddy soils. Environ. Res. 213, 113706. doi: 10.1016/j.envres.2022.113706, PMID: 35714686

[B133] Yu Y. Liu L. Wang J. Zhang Y. Xiao C. (2021b). Effects of warming on the bacterial community and its function in a temperate steppe. Sci. Total. Environ. 792, 148409. doi: 10.1016/j.scitotenv.2021.148409, PMID: 34146803

[B134] Yu H. Wang F. Shao M. Huang L. Xie Y. Xu Y. . (2021a). Effects of Rotations with legume on soil functional microbial communities involved in phosphorus transformation. Front. Microbiol. 12, 661100. doi: 10.3389/fmicb.2021.661100, PMID: 34659135 PMC8519609

[B135] Yuan M. M. Guo X. Wu L. Zhang Y. Xiao N. Ning D. . (2021). Climate warming enhances microbial network complexity and stability. Nat. Climate Change 11, 343–348. doi: 10.1038/s41558-021-00989-9

[B136] Zhai C. Han L. Xiong C. Ge A. Yue X. Li Y. . (2024). Soil microbial diversity and network complexity drive the ecosystem multifunctionality of temperate grasslands under changing precipitation. Sci. Total. Environ. 906, 167217. doi: 10.1016/j.scitotenv.2023.167217, PMID: 37751844

[B137] Zhang R. Chen L. Niu Z. Song S. Zhao Y. (2019). Water stress affects the frequency of Firmicutes, Clostridiales and Lysobacter in rhizosphere soils of greenhouse grape. Agric. Water Manage. 226, 105776. doi: 10.1016/j.agwat.2019.105776

[B138] Zhang B. Chen S. Zhang J. He X. Liu W. Zhao Q. . (2015). Depth-related responses of soil microbial communities to experimental warming in an alpine meadow on the Q inghai-T ibet P lateau. Eur. J. Soil Sci. 66, 496–504. doi: 10.1111/ejss.12240

[B139] Zhang C. Lei S. Wu H. Liao L. Wang X. Zhang L. . (2024). Simplified microbial network reduced microbial structure stability and soil functionality in alpine grassland along a natural aridity gradient. Soil Biol. Biochem. 191, 109366. doi: 10.1016/j.soilbio.2024.109366

[B140] Zhang Y. Li Y. Wang S. Umbreen S. Zhou C. (2021). Soil phosphorus fractionation and its association with soil phosphate-solubilizing bacteria in a chronosequence of vegetation restoration. Ecol. Eng. 164, 106208. doi: 10.1016/j.ecoleng.2021.106208

[B141] Zhang Z. Qiao M. Li D. Yin H. Liu Q. (2016). Do warming-induced changes in quantity and stoichiometry of root exudation promote soil N transformations via stimulation of soil nitrifiers, denitrifiers and ammonifiers? Eur. J. Soil Biol. 74, 60–68. doi: 10.1016/j.ejsobi.2016.03.007

[B142] Zhao M. Zhao J. Yuan J. Hale L. Wen T. Huang Q. . (2021). Root exudates drive soil-microbe-nutrient feedbacks in response to plant growth. Plant. Cell Environ. 44, 613–628. doi: 10.1111/pce.13928, PMID: 33103781

[B143] Zheng H. Liu Y. Chen Y. Zhang J. Li H. Wang L. . (2020). Short-term warming shifts microbial nutrient limitation without changing the bacterial community structure in an alpine timberline of the eastern Tibetan Plateau. Geoderma 360, 113985. doi: 10.1016/j.geoderma.2019.113985

